# Supplementary material for: Anticancer Activity of Water-Soluble Olsalazine-PAMAM-Dendrimer-Salicylic Acid-Conjugates
Source: Biomolecules. 2019 Aug 13;9(8):360. doi: 10.3390/biom9080360 (PMC6723055; doi:10.3390/biom9080360)
Supplement: Supplementary file 1 [file biomolecules-09-00360-s001.pdf]

Anticancer Activity of Water-Soluble Olsalazine-PAMAM-Dendrimer-Salicylic acid-Conjugates

Sandra Cortez-Maya[1], Luis Daniel Pedro-Hernández[1], Elena Martínez-Klimova[2], Teresa Ramírez-Ápan[1], Marcos Martínez-García[1*].

[1] Instituto de Química, Universidad Nacional Autónoma de México, Ciudad Universitaria, Circuito Exterior, Coyoacán, C.P. 04510, México D.F., México.

[2] Facultad de Química, Universidad Nacional Autónoma de México, Ciudad Universitaria, Circuito Interior, Coyoacán, C.P. 04510, México D.F., México.

Synthesis of olsalazine

A solution of mezalazine (6.00 g, 39 mM) in aqueous HCl (40 mL) was placed under vigorous stirring for 30 min. Then it was cooled to 0 °C. After 10 min $NaNO_2$ (13.47 g, 195 mmol) dissolved in water (25 mL) was added. The mixture was stirred for 30 min. The color of the solution changed from pink to pale yellow during the reaction. After that, salicylic acid (5.41 g, 39 mmol) diluted in 70 mL of aquous solution of 10% NaOH was added slowly. The mixture was stirred for 60 min at 0 °C. The mixture gave a yellow precipitate. The solid was filtered and washed with ethanol, yielding the compound **1** as a yellow solid (4.04 g, 13.38 mmol, 34%).

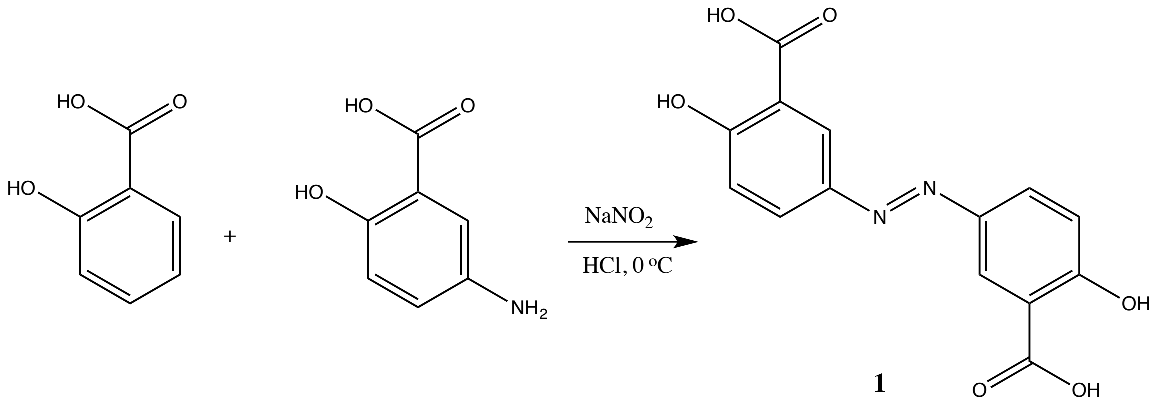

Scheme 1. Synthesis of olsalazine **1**.

UV-Vis (DMSO): $\lambda_{(nm)}$= 345. IR (KBr): ν(cm^{-1}) = 3426, 3081, 2182, 1709. ^{1}H NMR (400 MHz, DMSO-D$_6$): $\delta_{(ppm)}$= 6.73 (d, 2H, *J*= 9.5 Hz, Ar-3), 8.11 (dd, 2H, *J*= 9.5, 2.9 Hz, Ar), 8.89 (d, 2H, *J*= 2.9 Hz, Ar), 16.52 (br, 2H, COOH). ^{13}C NMR (100 MHz, DMSO-D$_6$): $\delta_{(ppm)}$= 87.7 (Ar-5), 120.0 (Ar), 124.5 (Ar), 135.4 (Ar), 139.5 (Ar), 166.3 (Ar), 180.7 (C=O). EM (IE+): m/z = 284. Elemen. Anal. calc. for. $C_{14}H_{10}N_2O_6$. C 55.64, H 3.34, N 9.27, O, 31.76 %. Found; C 55.64, H 3.33, N 9.27 %.

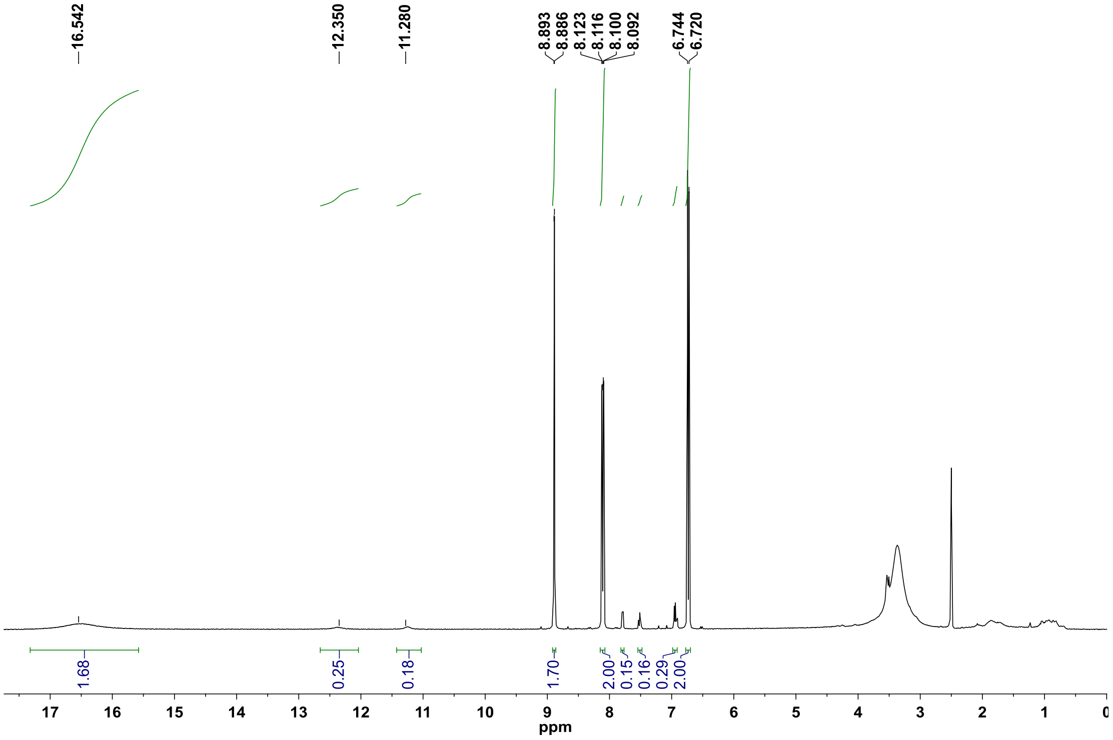

Figure 1. ^{1}H NMR spectrum of compound **1**.

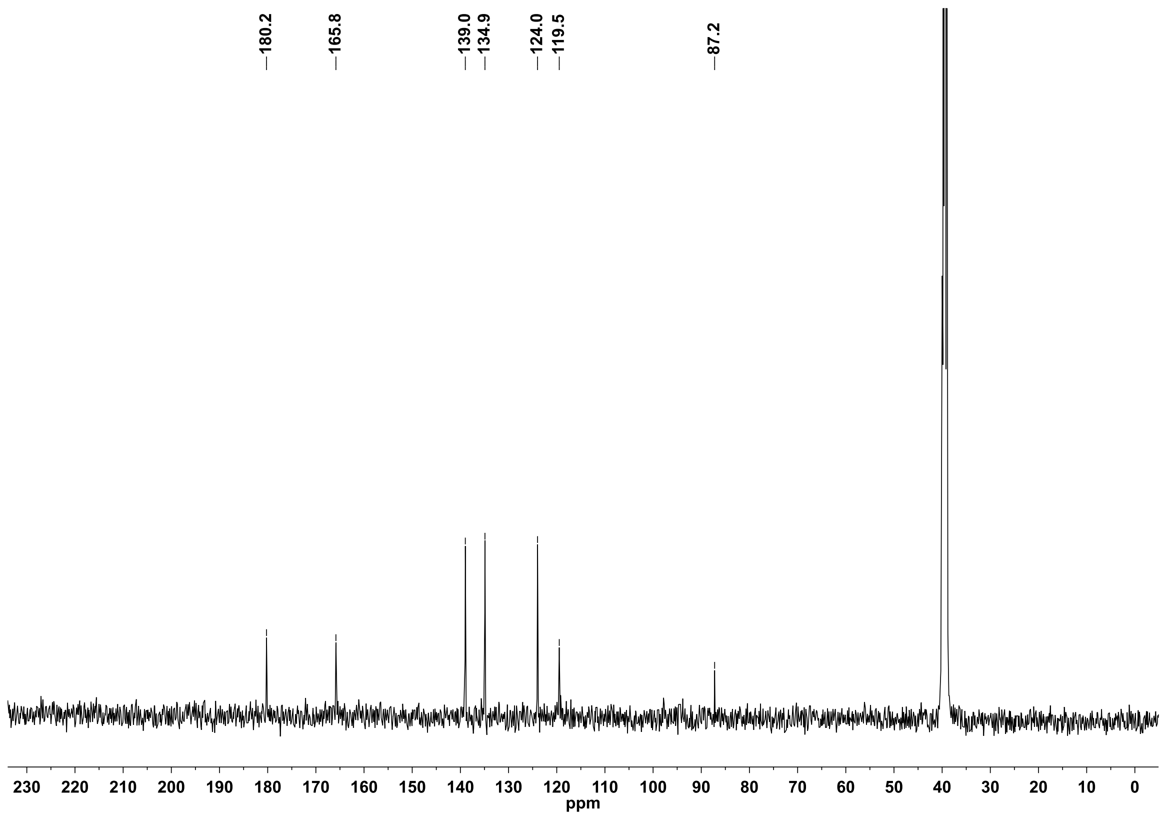

Figure 2. ^{13}C NMR spectrum of compound **1**.

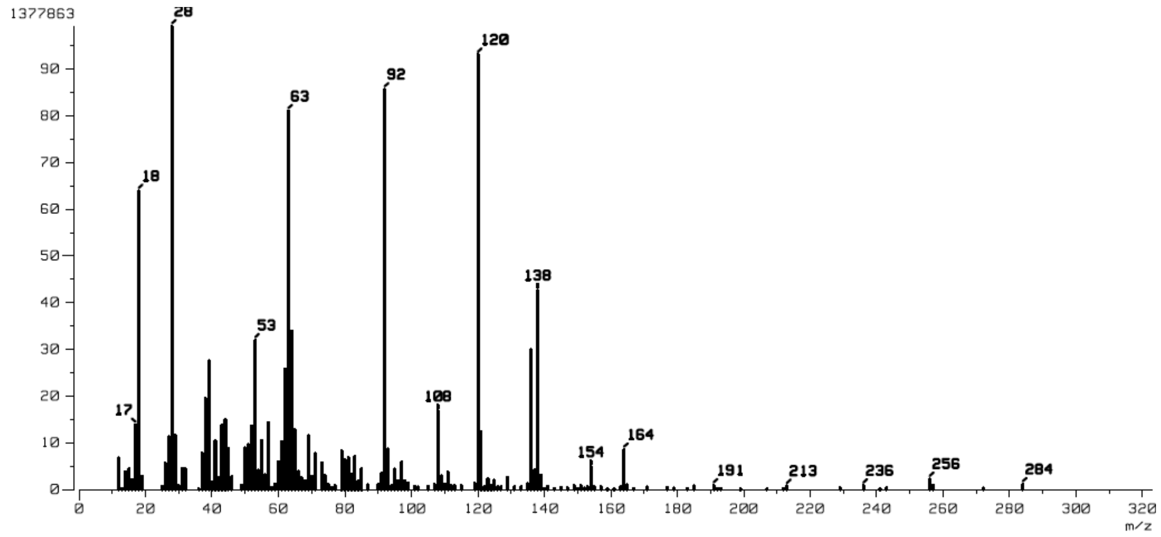

Figure 3. Mass spectrum of compound **1**.

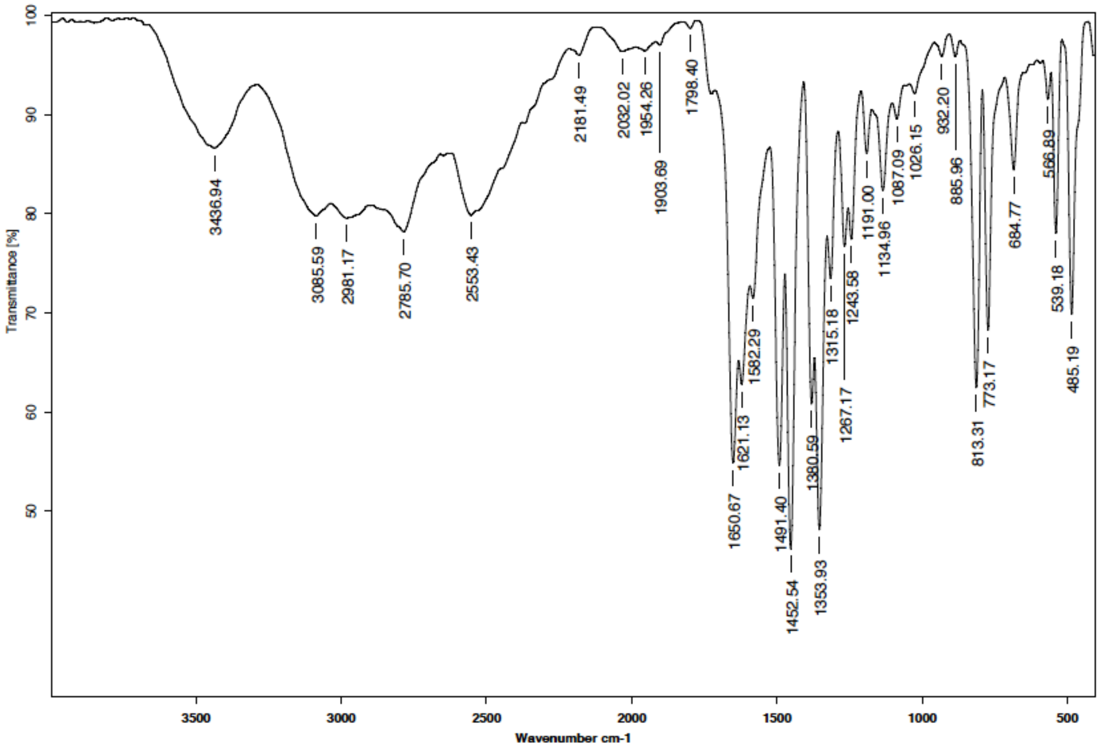

Figure 4. FTIR spectrum of compound **1**.

*Synthesis of the olsalazine derivative **2***

The compound **1** (1.00 g, 3.3 mmol) was dissolved in THF (15 mL) and molecular sieves of 4 Å (0.5 g) in N_2 atmosphere were stirred and heated at 70 °C for 10 min. After that, titanium(IV) isopropoxide (0.15 mL) was added. The mixture was stirred for 15 min and after that, hexemethylendiamine (0.77 g, 6.6 mmol) was added. Stirring was continued for 2 hours. Methanol was added (50 mL). The mixture was filtered and the solid was washed with methanol. The solvent was evaporated using a vacuum evaporator giving the compound **2** (1.315 g, 2.6 mmol, 79%) as a red oil.

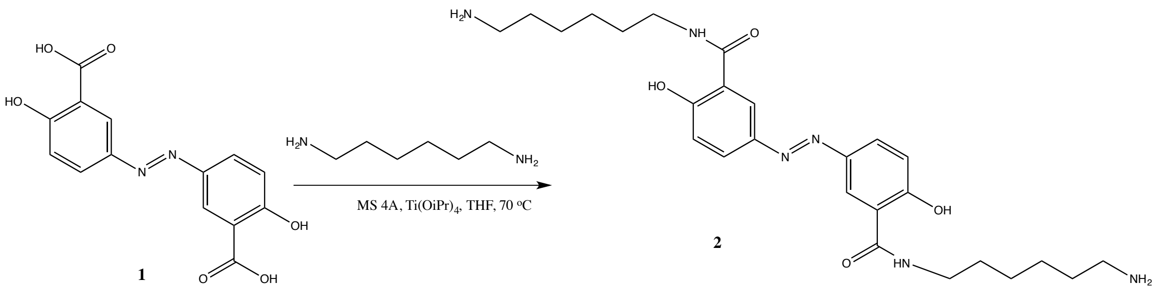

Scheme 2. Synthesis of the olsalazine derivative **2**.

UV-Vis (MeOH): $\lambda_{(nm)}$= 301, 213. IR (Film): $\nu(cm^{-1})$= 3296, 3074, 2936, 2862, 1635. ^{1}H NMR (300 MHz, CD_3OD): $\delta_{(ppm)}$= 1.23–1.42 (br, 4H, CH_2, 4H, NH_2), 1.49 (t, 4H, *J*= 5.7 Hz, CH_2), 1.63 (t, 4H, *J*= 5.6 Hz, CH_2), 1.79 (q, 2H, *J*= 6.5 Hz, CH_2), 2.25 (t, 2H, *J*= 7.5 Hz, CH_2), 2.89 (t, 2H, *J*= 7.19 Hz, CH_2-*NH_2*), 3.16 (t, 2H, *J= 6.9* Hz, CH_2-*NH_2*), 3.30 (q, 2H, *J*=1.5 Hz, CH_2-*NH*), 3.55 (t, 2H, *J*= 6.4 Hz, CH_2-*NH*), 6.63 (t, 2H, *J*= 8.7 Hz, NH)6.79 (d, 2H, *J*= 8.5 Hz, Ar), 7.26 (d, 2H, *J*= 2.9 Hz, Ar), 7.83 (dd, 2H, *J*= 7.7, 1.6, Ar). ^{13}C NMR (75 MHz, CD_3OD): $\delta_{(ppm)}$= 27.0 (CH_2), 27.3 (CH_2), 27.6 (CH_2), 28.5 (CH_2), 29.9 (CH_2), 30.1 (CH_2), 30.3 (CH_2), 30.7 (CH_2), 33.7 (CH_2-*NH_2*), 40.0 (CH_2-*NH_2)*, 40.2 (CH_2-*NH*), 40.6 (CH_2-*NH*), 117.1(Ar-1), 119.0 (Ar-3), 131.6 (Ar-6), 133.8 (Ar-4), 162.6 (Ar-5), 163.8 (Ar-2), 175.9 (C=O). EM DART: m/z = 498. Elemen. Anal. calc. for. $C_{26}H_{38}N_6O_4$. C 62.63, H 7.68, N 16.85, O 12.83 %. Found: C 62.61, H 7.66, N 16.83 %.

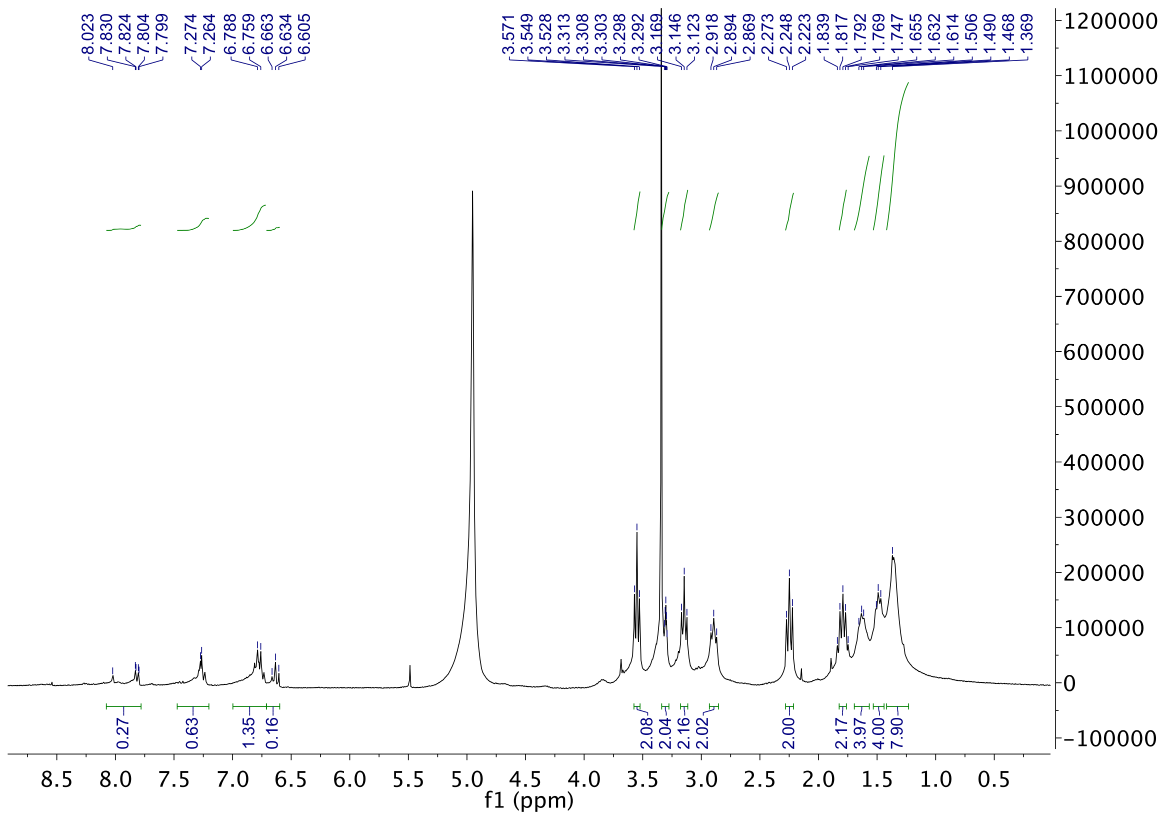

Figure 5. ^{1}H NMR spectrum of compound **2**.

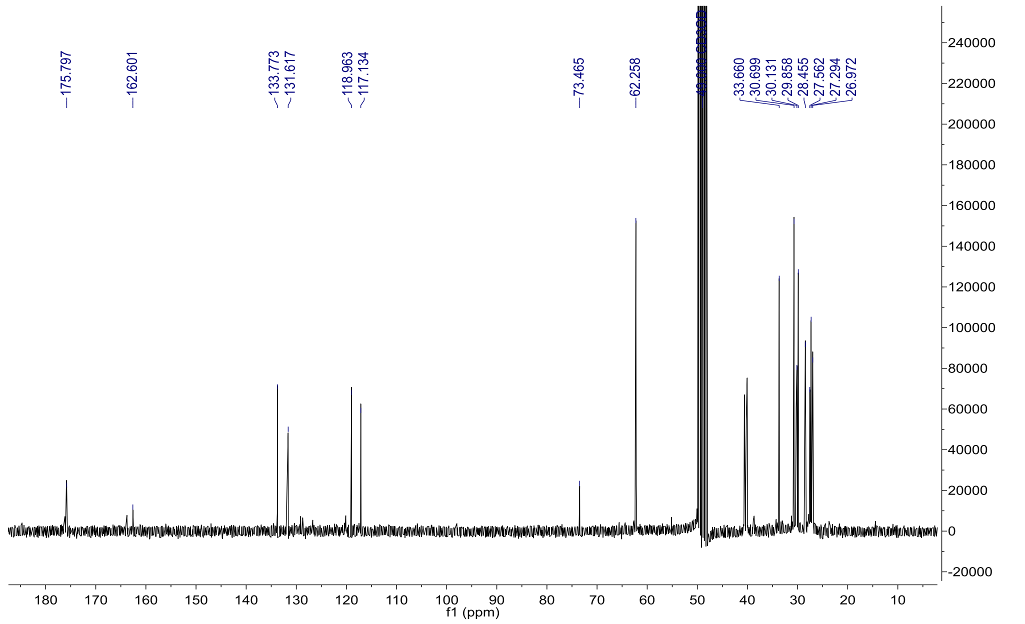

Figure 6. ^{13}C NMR spectrum of compound **2**.

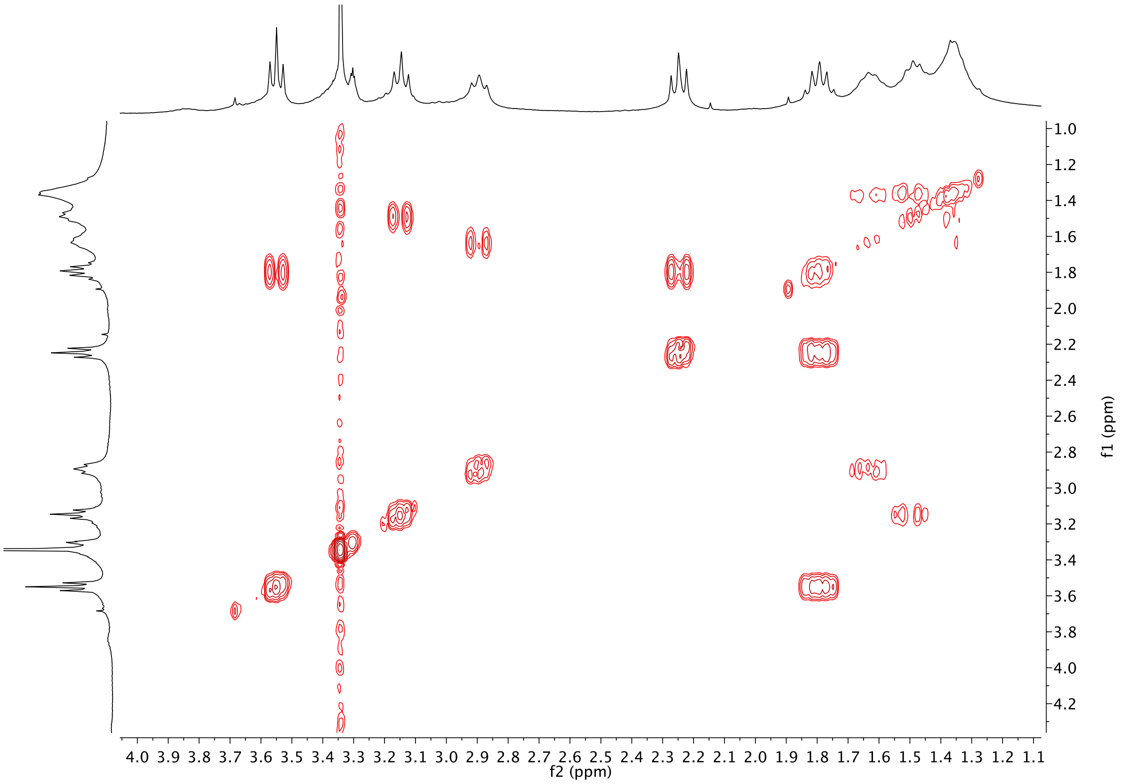

Figure 7. COSY ^{1}H-^{1}H NMR spectrum of compound **2** in the aliphatic region.

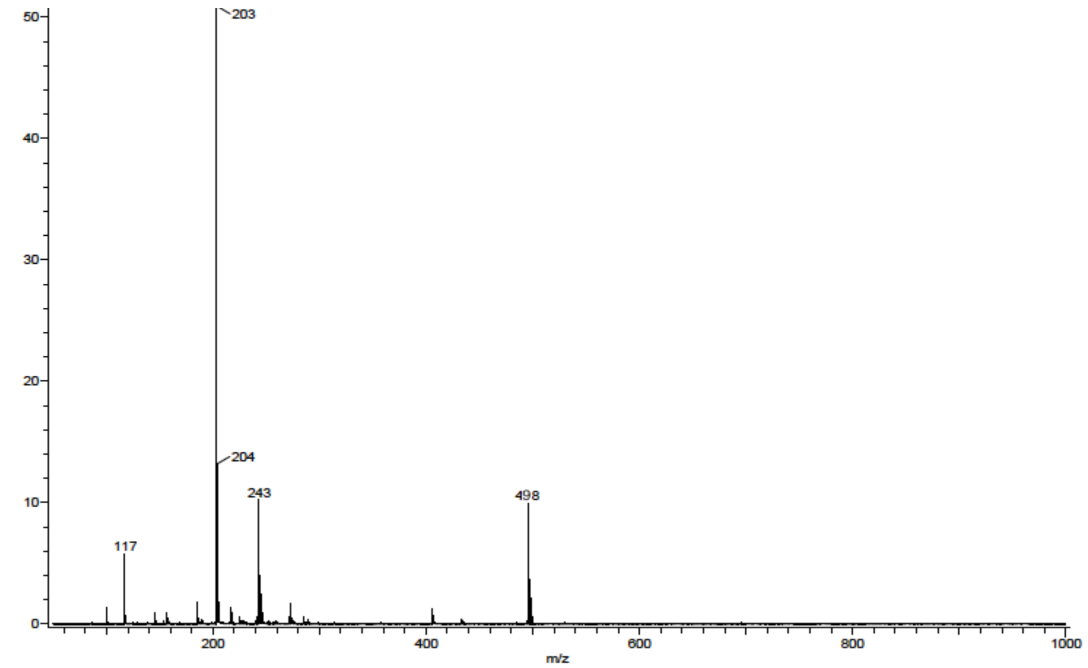

Figure 8. Mass spectrum of compound **2**.

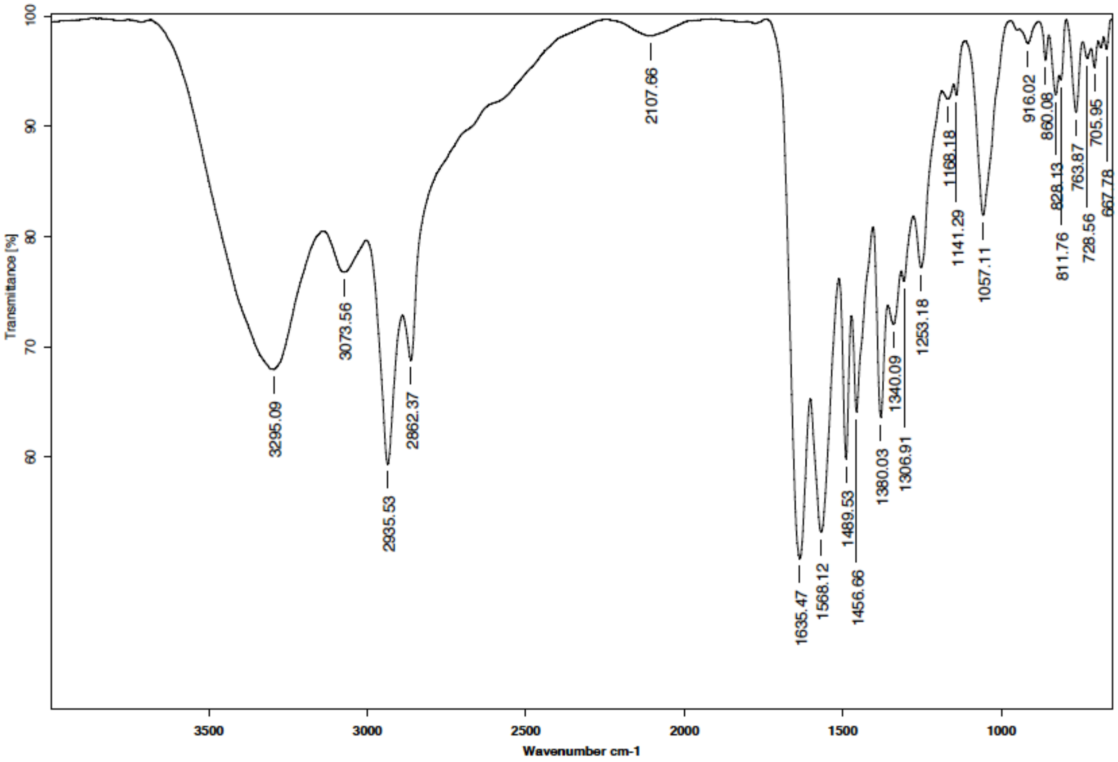

Figure 9. FTIR spectrum of compound **2**.

General procedures for the synthesis of olsalazine-PAMAM-intermediates 3 and 5.

Compound **2** or **4** was dissolved in methanol and heated to 80°C. After 20 min, methyl acrylate was added in methanol. The mixture was stirred and heated at 120 °C for 24 h. The solvent was evaporated and the resulting solid was dissolved in methanol and precipitated by EtOAc.

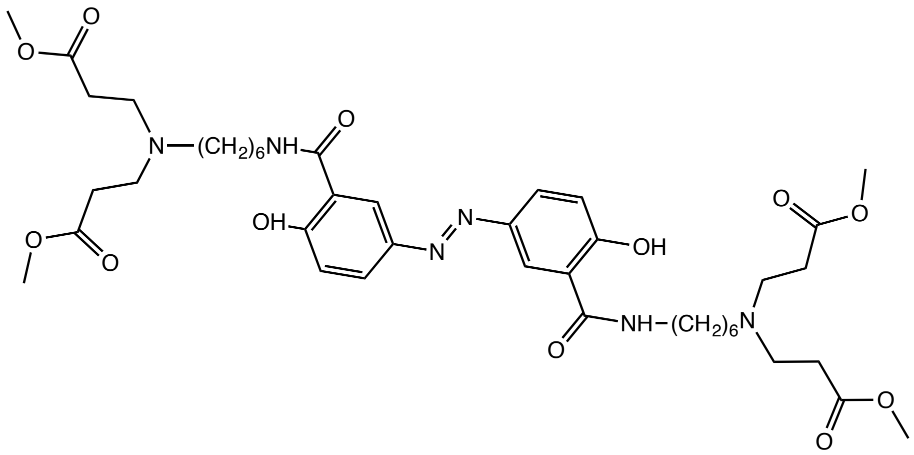

Scheme 3. Synthesis of olsalazine-PAMAM-intermediates 3 and 5

Compound 3.

Compound **2** (0.650 g, 1.3 mmol) methyl acrylate (1.8 mL, 39 mmol). Red liquid. Yield (1.087 g, 1.3 mmol, 99%). UV-Vis (MeOH): $\lambda_{(nm)}$= 298, 210. IR (Film): $\nu_{(cm^{-1})}$= 3378, 2938, 2860, 1733, 1632. ^{1}H NMR (300 MHz, CD_3OD): $\delta_{(ppm)}$= 1.40 (br, 4H, CH_2), 1.52 (q, 4H, *J*= 7.0 Hz, CH_2), 1.69 (q, 4H, *J*= 7.4 Hz, CH_2), 1.81 (q, 2H, *J*= 6.6 Hz, CH_2), 2.26 (t, 2H, *J*= 7.2 Hz, CH_2), 2.46 (t, 2H, *J*= 6.9 Hz, CH_2-*N*), 2.59 (t, 2H, *J*= 6.7 Hz, CH_2-*N*), 2.78 (t, 4H, *J*= 6.7 Hz, CH_2-*C=O*), 2.96 (t, 2H, *J*= 7.8 Hz, CH_2), 3.22 (m, 6H, CH_2-*N*, CH_2), 3.34 (s, 4H, CH_2), 3.56 (t, 4H, *J* = 6.4 Hz, CH_2), 3.67–3.76 (m, 12H, CH_3), 6.76 - 6.80 (d, 2H, *J*= 8.5 Hz, Ar-3), 7.24–7.34 (m, 2H, Ar-6), 7.83–7.89 (dd, 2H, *J*= 7.7, 1.8 Hz, Ar-4), 8.03 (br, 1H, NH), 8.31 (br, 1H, NH). ^{13}C NMR (125 MHz, CD_3OD): $\delta_{(ppm)}$= 27.0 (CH_2), 27.1 (CH_2), 27.3 (CH_2), 27.7 (CH_2), 29.9 (CH_2), 30.1 (CH_2), 30.3 (CH_2), 31.3 (CH_2), 33.7 (CH_2-*N*), 34.7 (CH_2-*N*), 40.0 (CH_2-*NH*), 40.3 (CH_2-*NH*), 42.3 (CH_2-*C=O*), 44.3 (CH_2-*C=O*), 50.3 (CH_2-*N*), 52.1 (CH_2-*N*), 52.7 (CH_3), 117.3 (Ar-1),

119.1 (Ar-3), 131.7 (Ar-6), 134.2 (Ar-4), 155.4 (Ar-5), 162.6 (Ar-2), 175.9 (C=O), 177.4 (C=O). EM FAM+): m/z = 843 $[M]^{+}$. Elemen. Anal. calc. for. $C_{42}H_{62}N_{6}O_{12}$. C 59.84.63, H 7.41, N 9.97, O 22.77 %. Found: C 59.86, H 7.43, N 9.98 %.

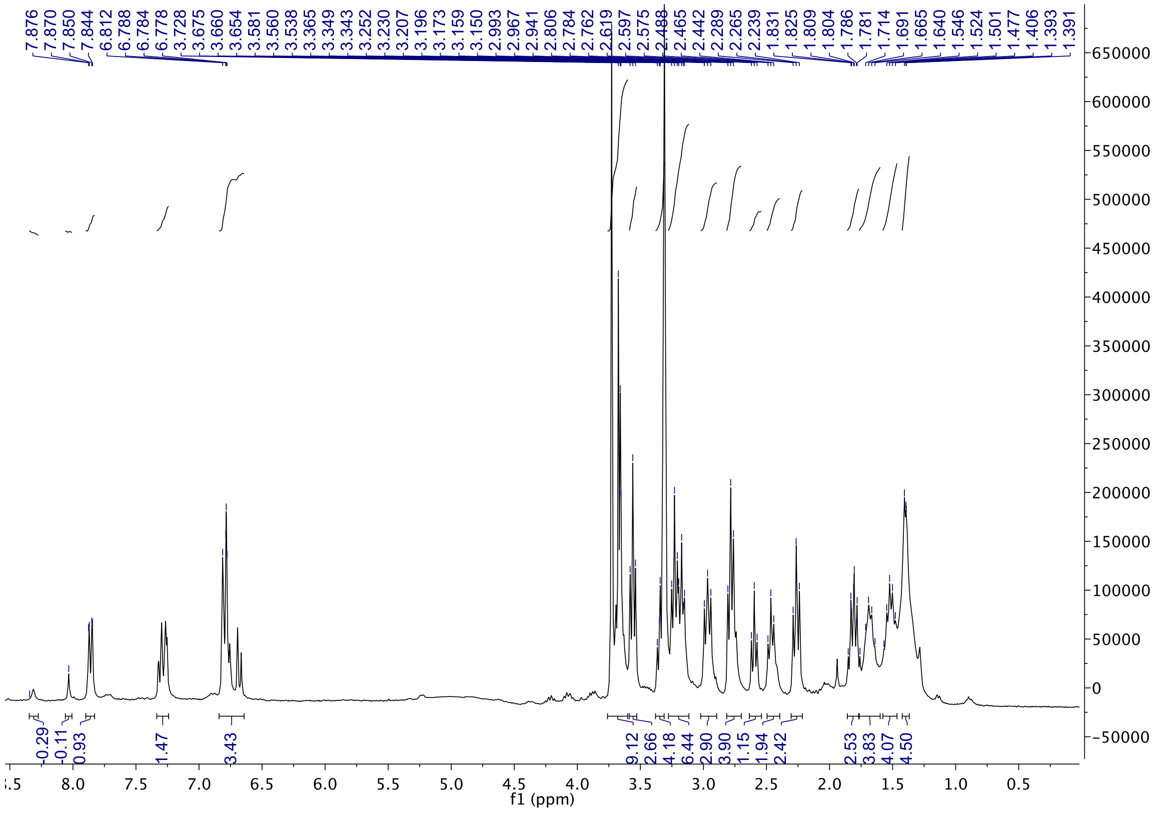

Figure 10. ^{1}H NMR spectrum of compound **3**.

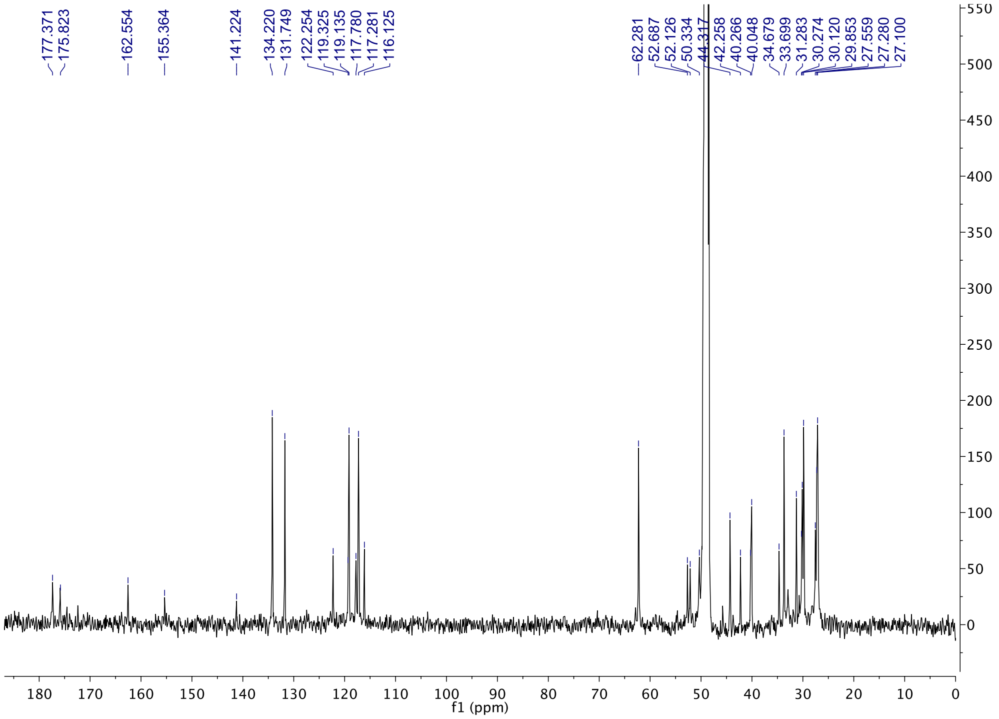

Figure 11. ^{13}C NMR spectrum of compound **3**.

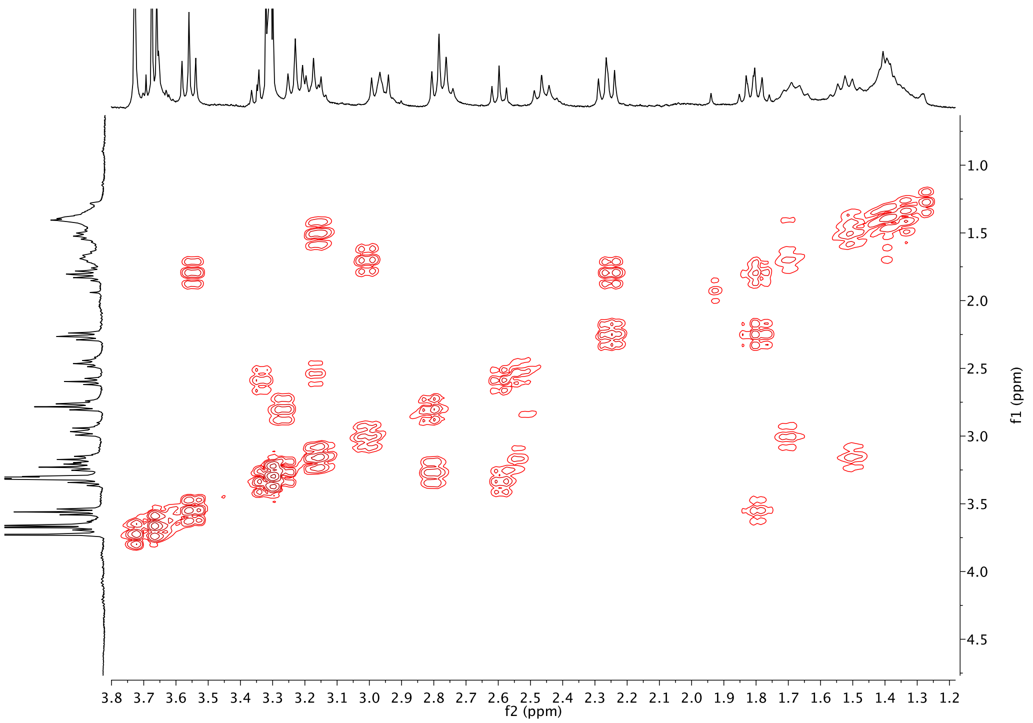

Figure 12. COSY NMR spectrum of compound **3** in the aliphatic region.

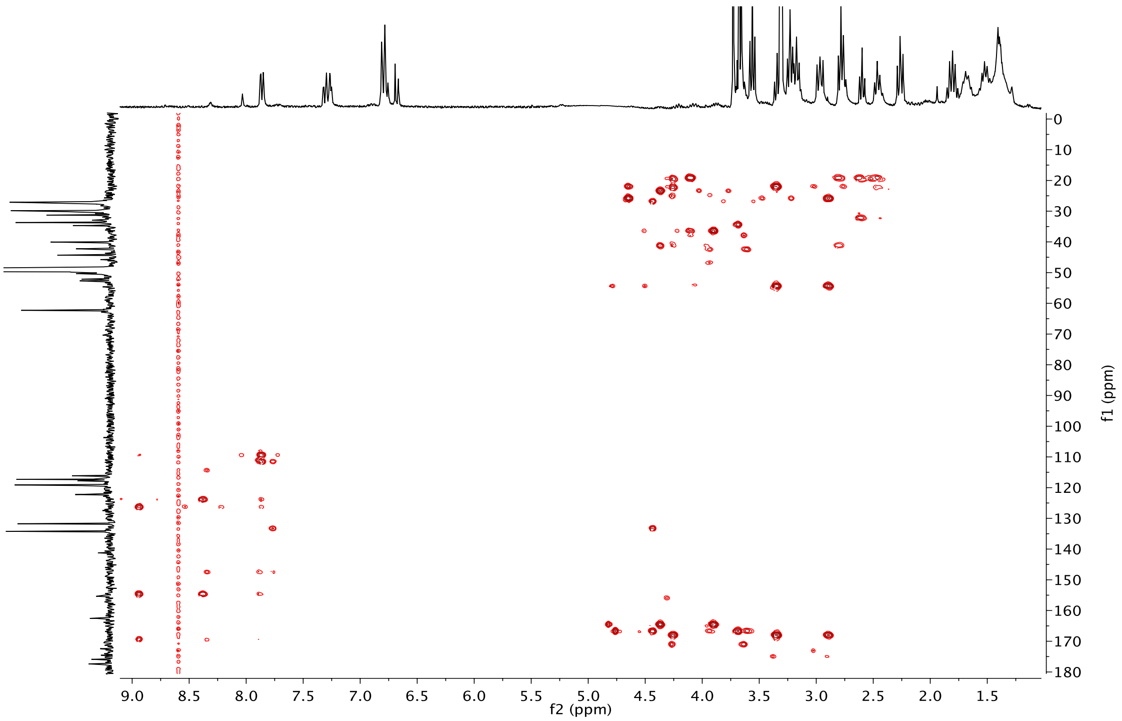

Figure 13. HMBC NMR spectrum of compound **3**.

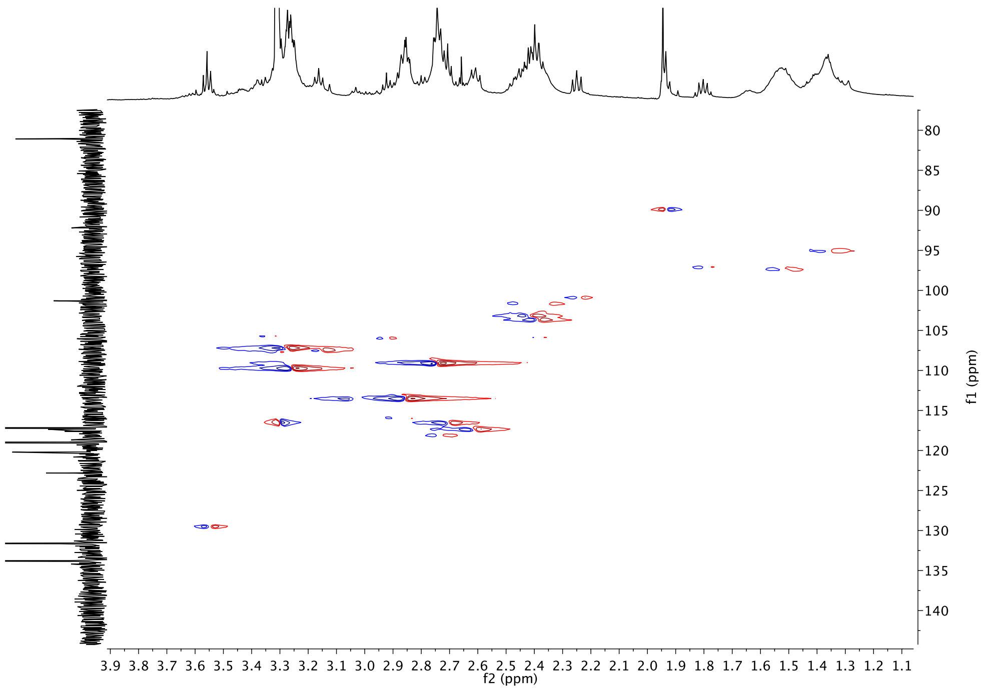

Figure 14. HSQC NMR spectrum of compound **3**.

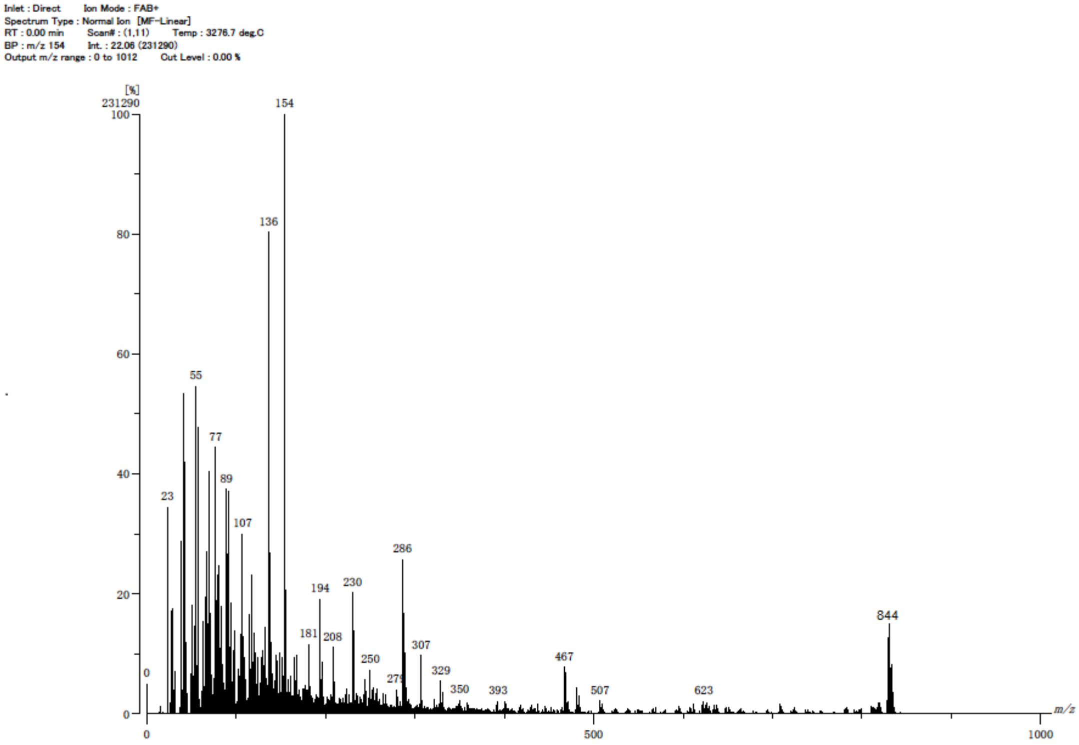

Figure 15. FAB+ mass spectrum of compound **3**.

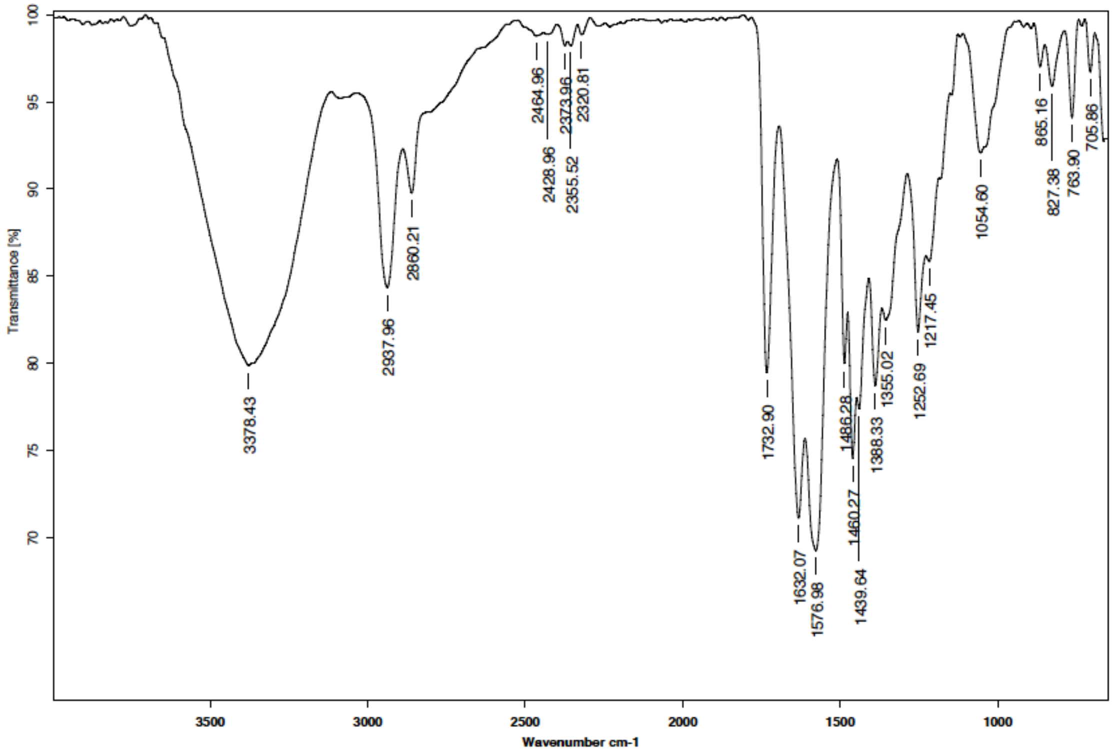

Figure 16. FTIR spectrum of compound **3**.

Compound 5.

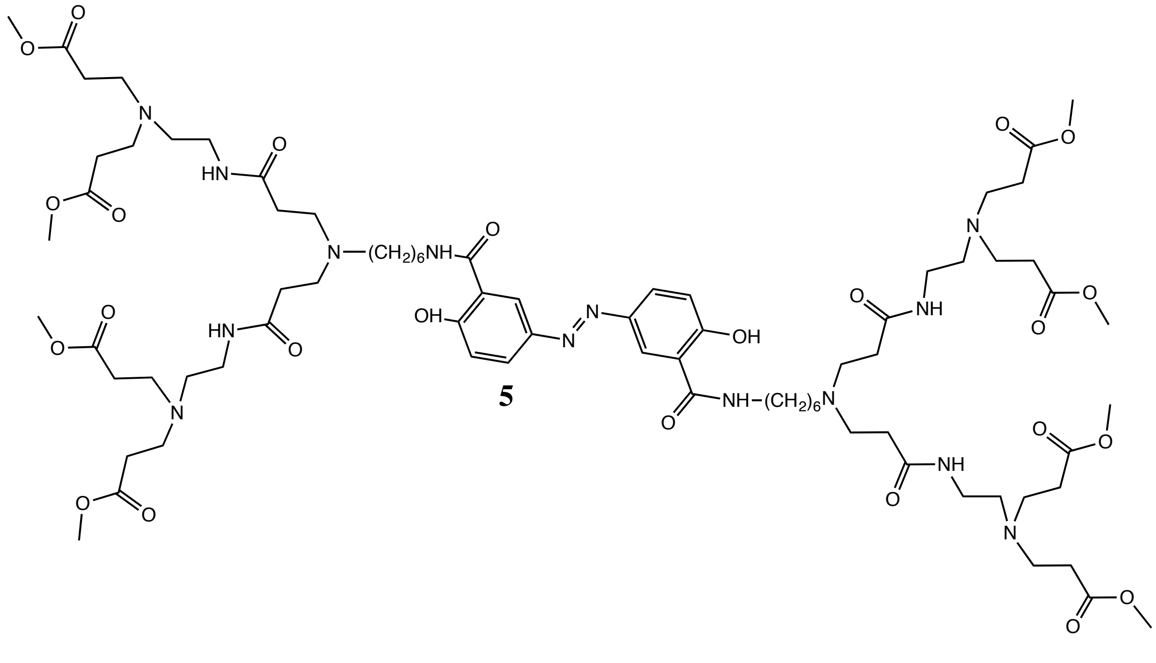

Compound **4** (0.260 g, 0.272 mmol) methyl acrilate (0.6 mL, 39 mmol). Red liquid. Yield (0.439 g, 0.267 mmol, 98%). UV-Vis (MeOH): $\lambda_{(nm)}$= 207. IR (Film): $\nu_{(cm^{-1})}$= 3370, 3081, 2952, 2845, 1735, 1651. ^{1}H NMR (500 MHz, CD_3OD): $\delta_{(ppm)}$= 1.34 (br, 6H, CH_2), 1.49 (br, 6H, CH_2), 2.25 (t, 1H, *J*= 4.5, CH_2), 2.35 (m, 6H, CH_2-*N*), 2.46 (br, 26H, CH_2-*C=O*), 2.47 (br, 26H, CH_2-*NH*), 2.55 (br, 8H, CH_2-*N*), 2.77 (br, 28H, CH_2-*N*), 3.16 (t, 2H, *J*= 3.9 Hz, CH_2), 3.27 (s, 6H, CH_2), 3.56 (t, 1H, *J*= 3.9 Hz, CH_2), 3.67 (s, 24H, O-CH_3), 6.58–6.97 (m, Ar-3), 7.19–7.48 (m, Ar-6), 7.93–8.13 (m, Ar-4). ^{13}C NMR (125 MHz, CD_3OD): $\delta_{(ppm)}$= 27.4 (CH_2), 27.9 (CH_2), 28.2 (CH_2), 28.6 (CH_2), 29.9 (CH_2), 30.4 (CH_2), 33.3 (CH_2-*N*), 33.6 (CH_2-*N*), 34.5 (CH_2-*NH*), 34.7 (CH_2-*NH*), 38.4 (CH_2-*C=O*), 40.3 (CH_2-*C=O*), 45.5 (CH_2-*N*), 45.6 (CH_2-*N*), 46.4 (CH_2-*NH*), 47.6 (CH_2-*NH*), 50.5 (CH_2-*N*), 52.1 (CH_2-*N*), 53.7 (CH3), 117.4 (Ar-1), 120.4 (Ar-3), 129.3 (Ar-6), 121.5 (Ar-4), 163.7 (Ar-5), 168.2 (Ar-2), 173.1 (C=O), 174.7 (C=O), 175.7 (C=O). EM FAB+): m/z = 1643 $[M]^{+}$. Elemen. Anal. calc. for. $C_{78}H_{126}N_{14}O_{24}$. C 56.99, H 7.73, N 11.93, O 23.36 %. Found: C 56.96, H 7.71, N 11.95 %.

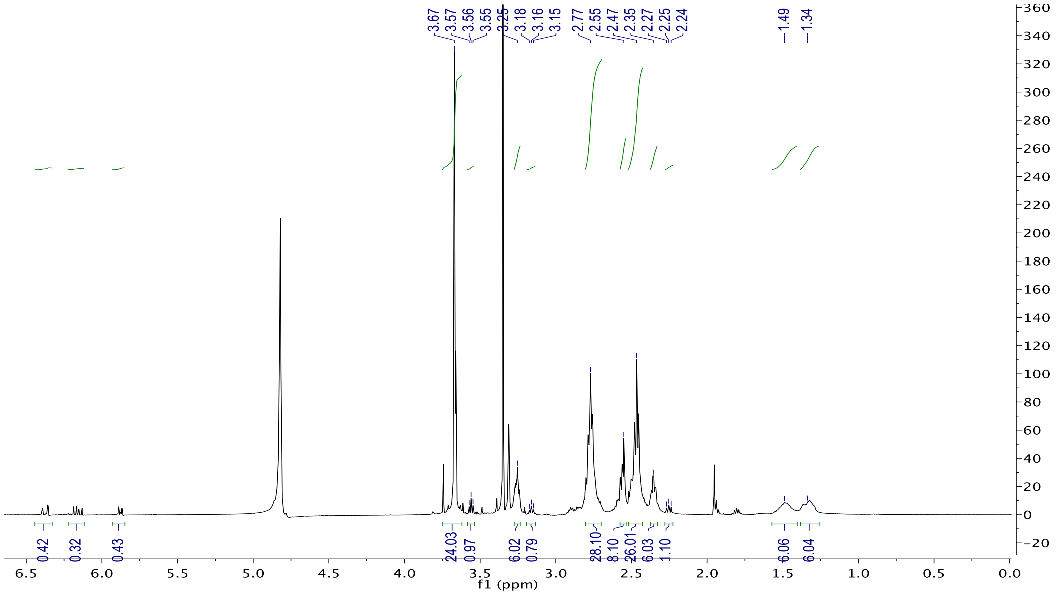

Figure 17. ^{1}H NMR spectrum of compound **5**.

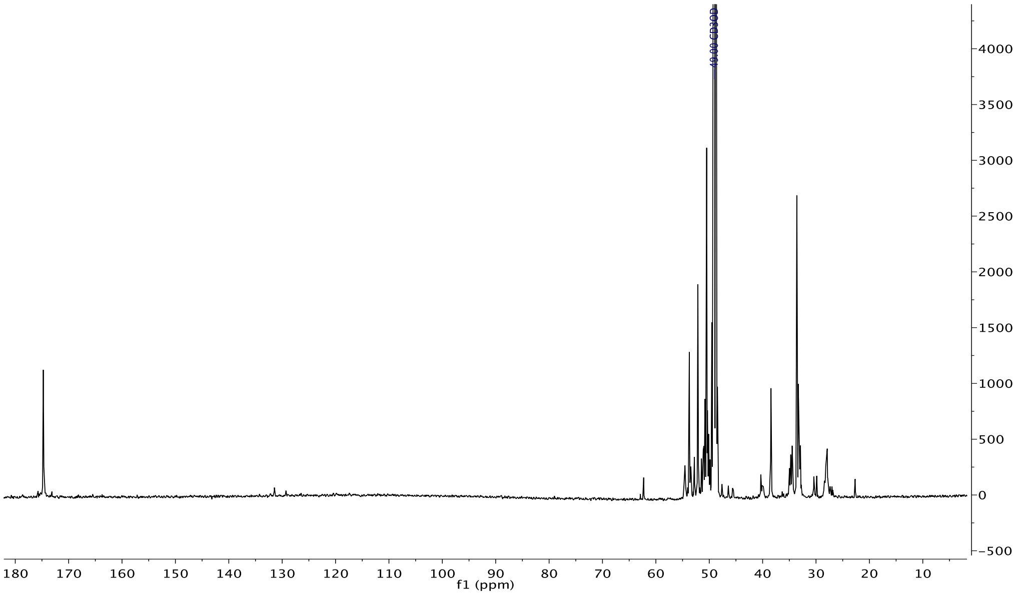

Figure 18. ^{13}C NMR spectrum of compound **5**.

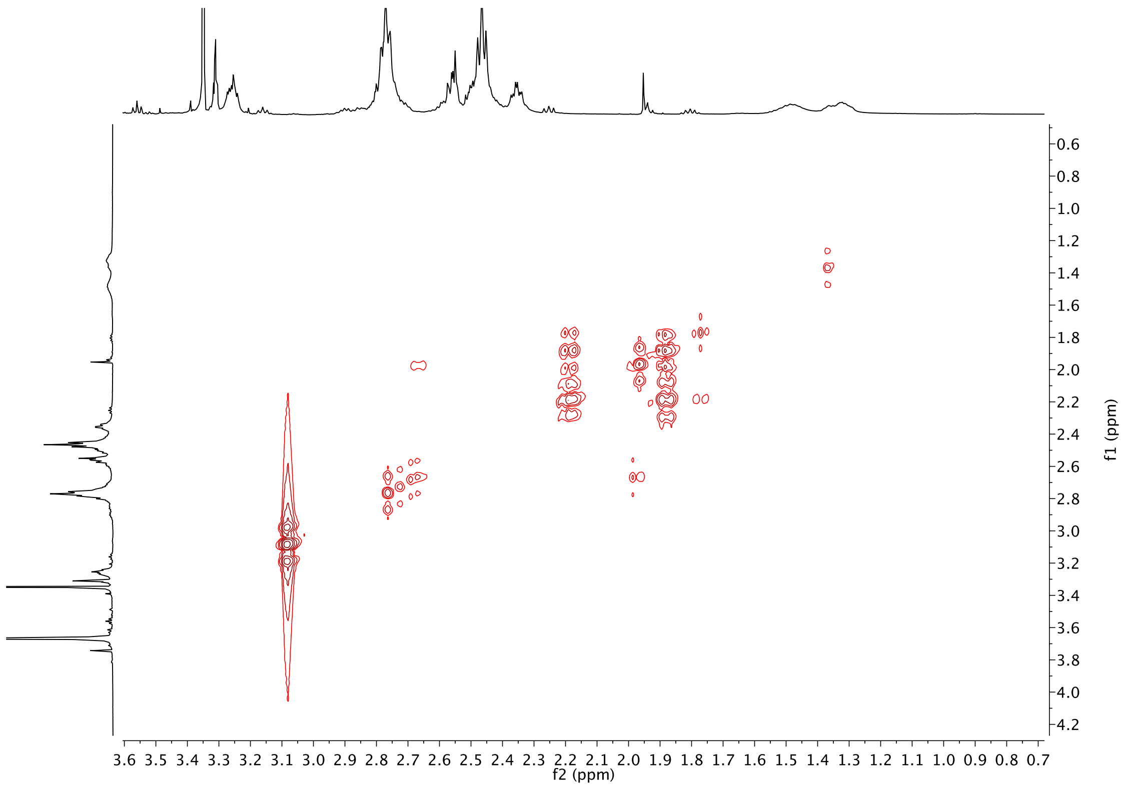

Figure 19. COSY NMR spectrum of compound **5**.

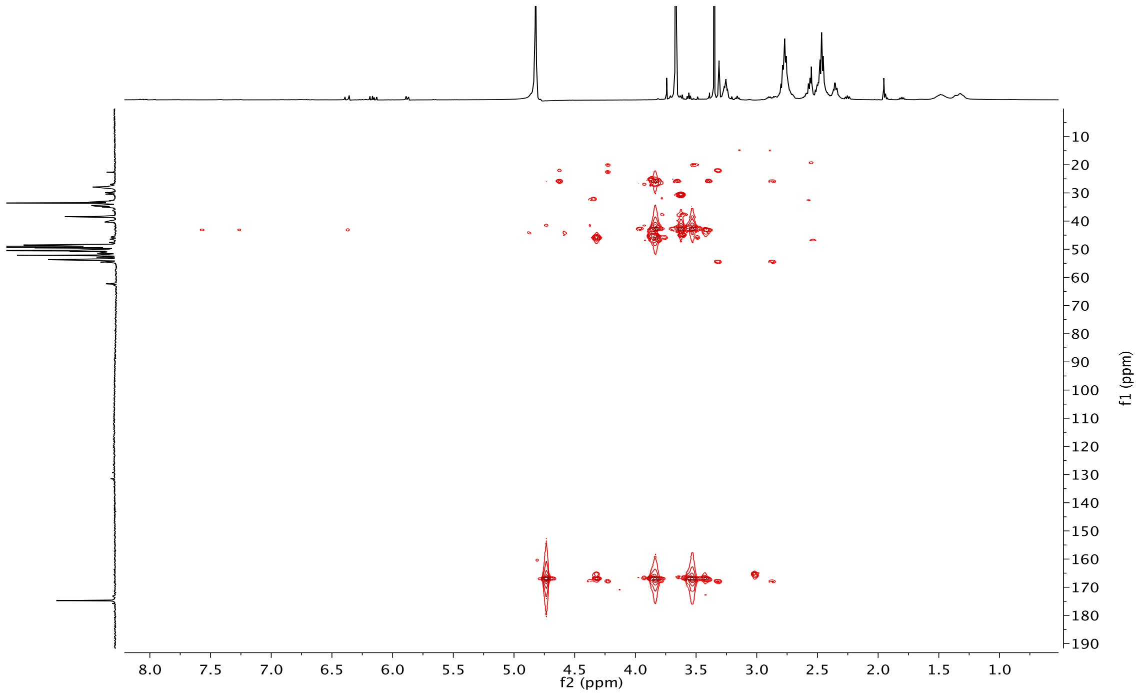

Figure 20. HMBC NMR spectrum of compound **5**.

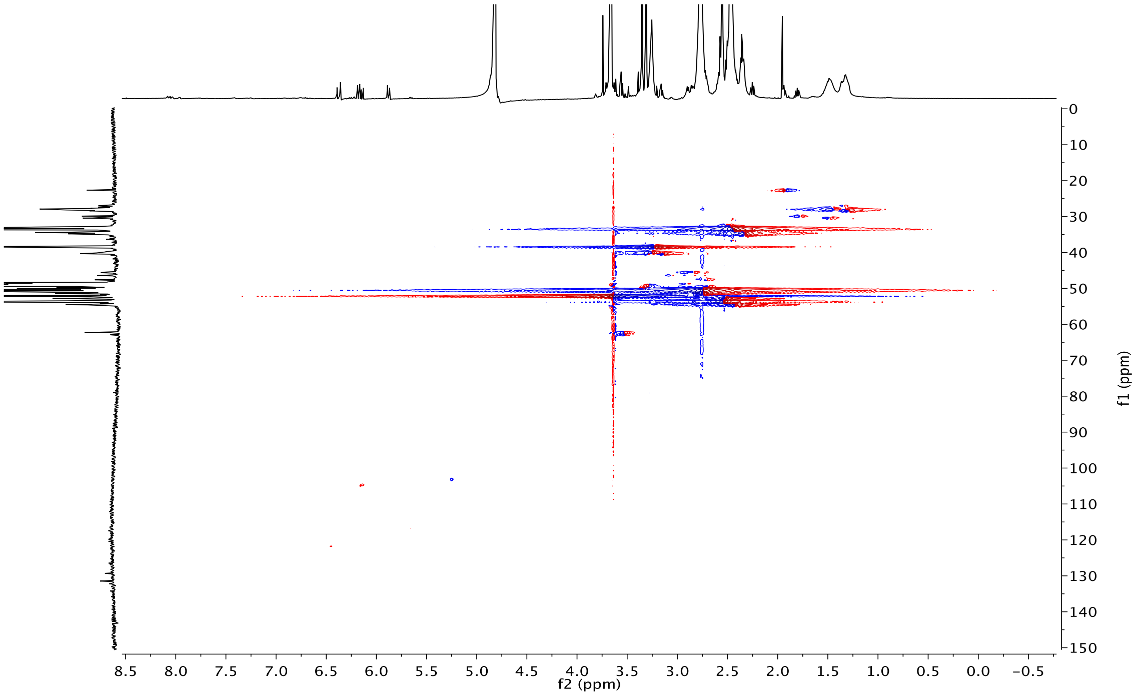

Figure 21. HSQC NMR spectrum of compound **5**.

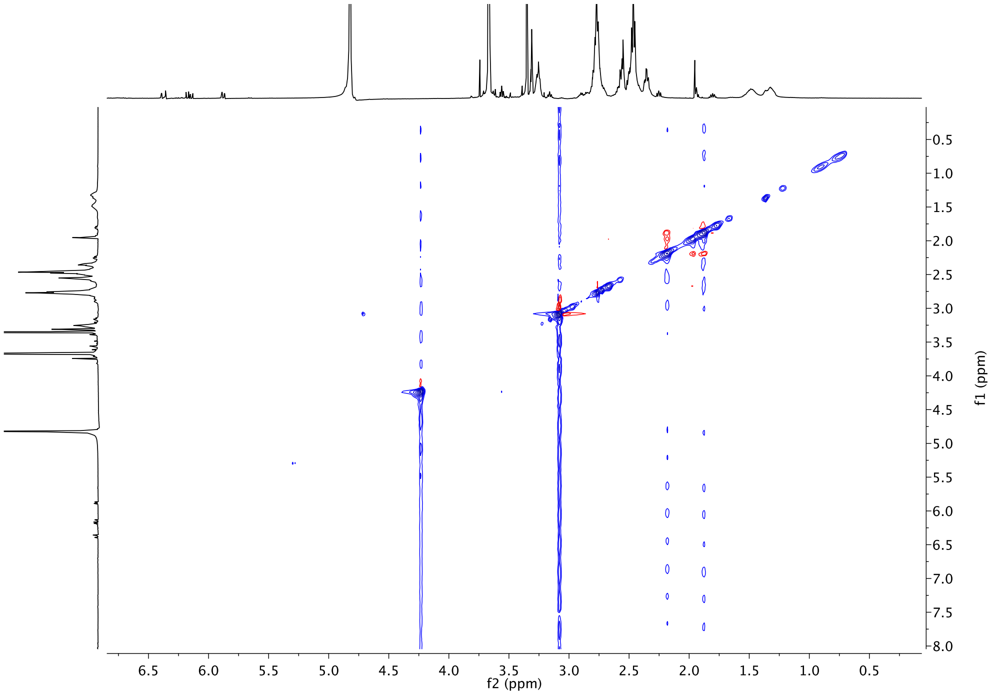

Figure 22. NOESY NMR spectrum of compound **5**.

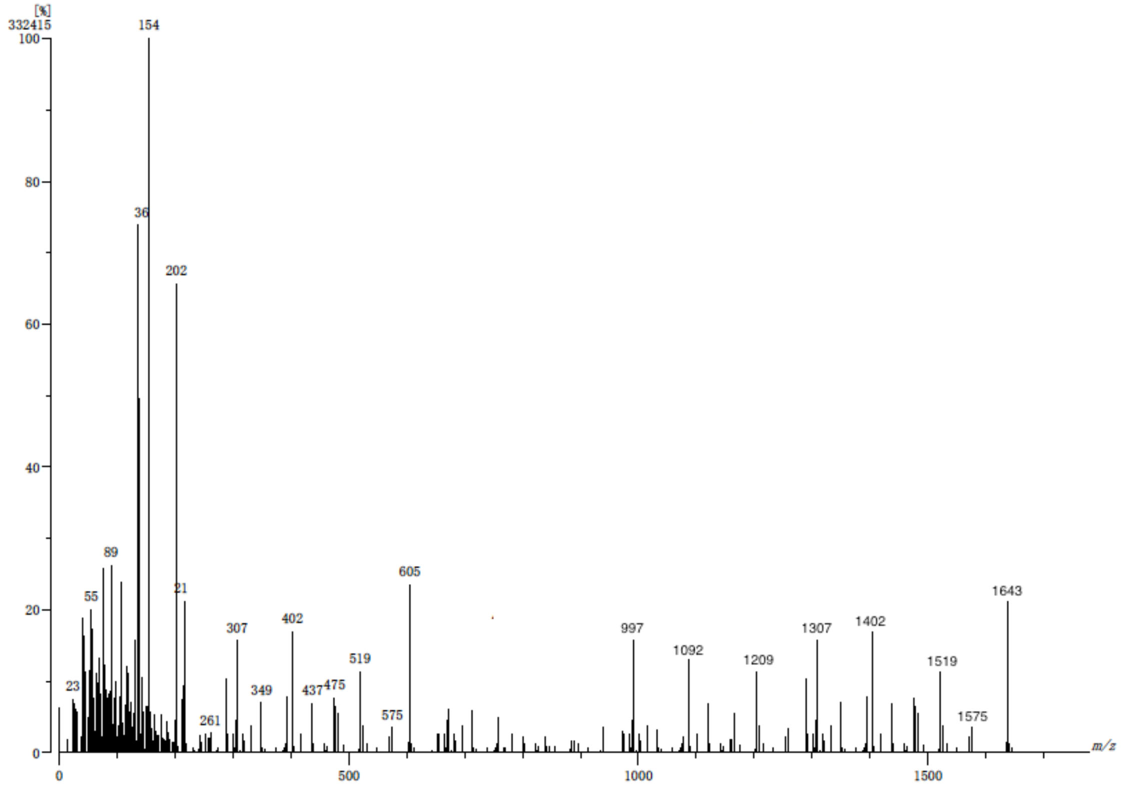

Figure 23. Mass spectrum of compound **5**.

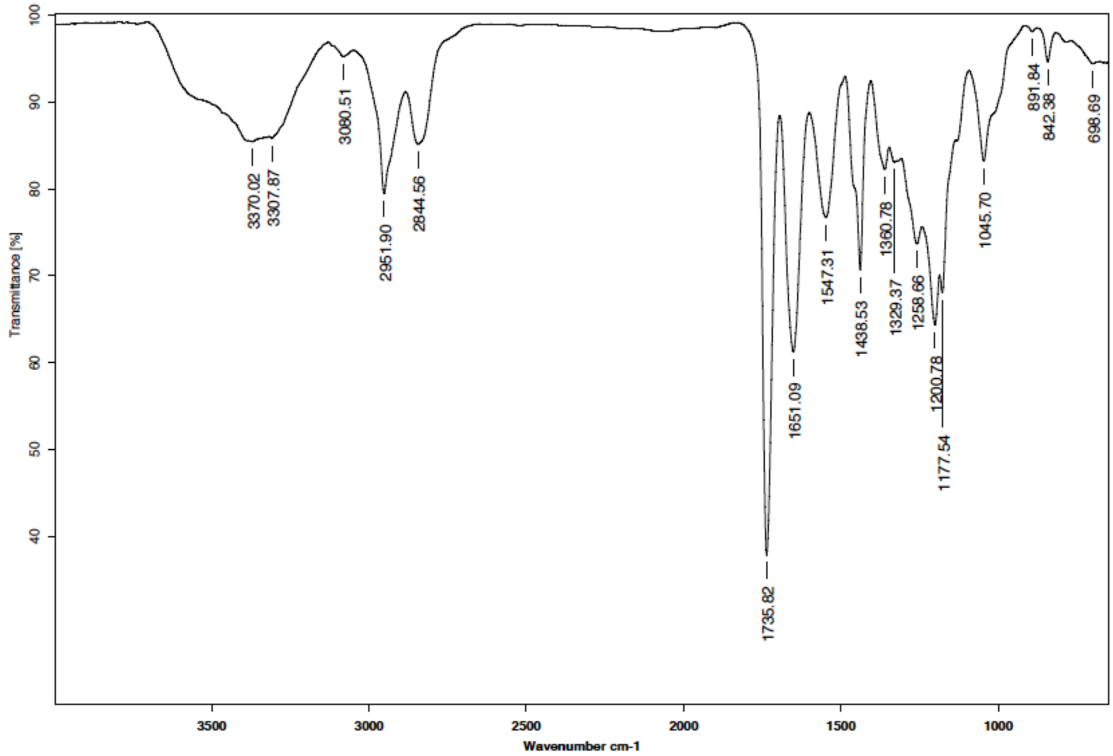

Figure 24. FTIR spectrum of compound **5**.

General procedures for the synthesis of olsalazine-PAMAM-dendrimers 4 and 6.

Compound **3** or **5** (0.0525 mmol) was dissolved in methanol (40 mL) and heated to 80°C. After 20 min, methyl acrylate (0.63 mmol) was added in methanol. The mixture was stirred and heated at 120 °C for 24 h. The solvent was evaporated and the resulting solid was dissolved in methanol and precipitated by EtOAc.

Compound 4.

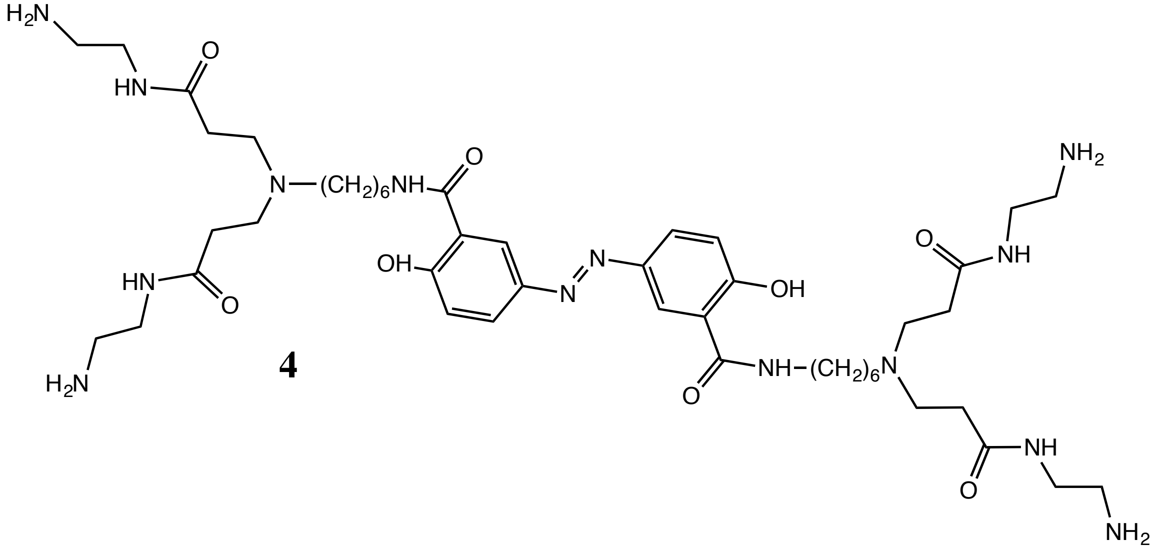

4

Compound **3** (1.087 g, 1.3 mmol) ethylenediamine (1.3 mL, 19.5 mmol). Red liquid, Yield (1.270 g, 1.3 mmol, 99%). UV-Vis (MeOH): $\lambda_{(nm)}$= 207. IR (Film $\nu_{(cm^{-1})}$= 3384, 3097, 2940, 2864, 1644. ^{1}H NMR (500 MHz, CD_3OD): $\delta_{(ppm)}$= 1.25–1.45 (br, 12H, NH_2, CH_2), 1.48–1.59 (br, 6H, CH_2), 1.60–1.70 (br, 2H, CH_2), 1.80 (q, 2H, *J*= 6.5 Hz, CH_2), 1.93–1.95 (m, 2H, CH_2), 2.25 (t, 2H, *J*= 7.5 Hz, CH_2-*N*), 2.37–2.44 (m, 8H, CH_2-*C=O*), 2.61 (t, 2H, *J*= 7.4 Hz, CH_2-*N*), 2.71–2.77 (m, 8H, CH_2-*N*), 2.82–2.90 (m, 8H, CH_2-*NH_2*), 2.92 (t, 2H, *J*= 6.6 Hz, NH), 3.16 (t, 4H, *J*= 7.0 Hz, NH), 3.23–3.39 (m, 8H, CH_2-*NH*), 3.33–3.37 (m, 2H, CH_2-*NH*), 3.56 (t, 2H, *J*= 6.6 Hz, CH_2-*NH*), 6.57–6.82 (m, Ar-3), 7.22–7.43 (m, Ar-6), 8.00 8.11 (m, Ar-4). ^{13}C NMR (75 MHz, CD_3OD): $\delta_{(ppm)}$= 27.5 (CH_2), 27.8 (CH_2), 27.9 (CH_2), 28.1 (CH_2), 29.9 (CH_2), 30.1 (CH_2), 30.2 (CH_2), 30.3 (CH_2), 33.7 (CH_2-*N*), 34.4 (CH_2-*N*), 40.4 (CH_2-*NH*), 40.7 (CH_2-*NH*), 41.8 (CH_2-*C=O*), 42.5 (CH_2-*C=O*), 44.4 (CH_2-*NH_2*), 44.9 (CH_2-*NH2*), 45.0 (CH_2-*NH*), 46.4 (CH_2-*NH*), 50.6 (CH_2-*N*), 117.6 (Ar-1), 119.0 (Ar-3), 131.6 (Ar-6), 133.8 (Ar-4), 162.6 (Ar-5), 169.7

(Ar-2), 176.1 (C=O), 180.6 (C=O). EM FAB+: m/z = 954 $[M]^+$. Elemen. Anal. calc. for. $C_{46}H_{78}N_{14}O_8$. C 57. 84, H 8.23, N 20.53, O 13.40 %. Found: C 57.87, H 8.21, N 20.50 %.

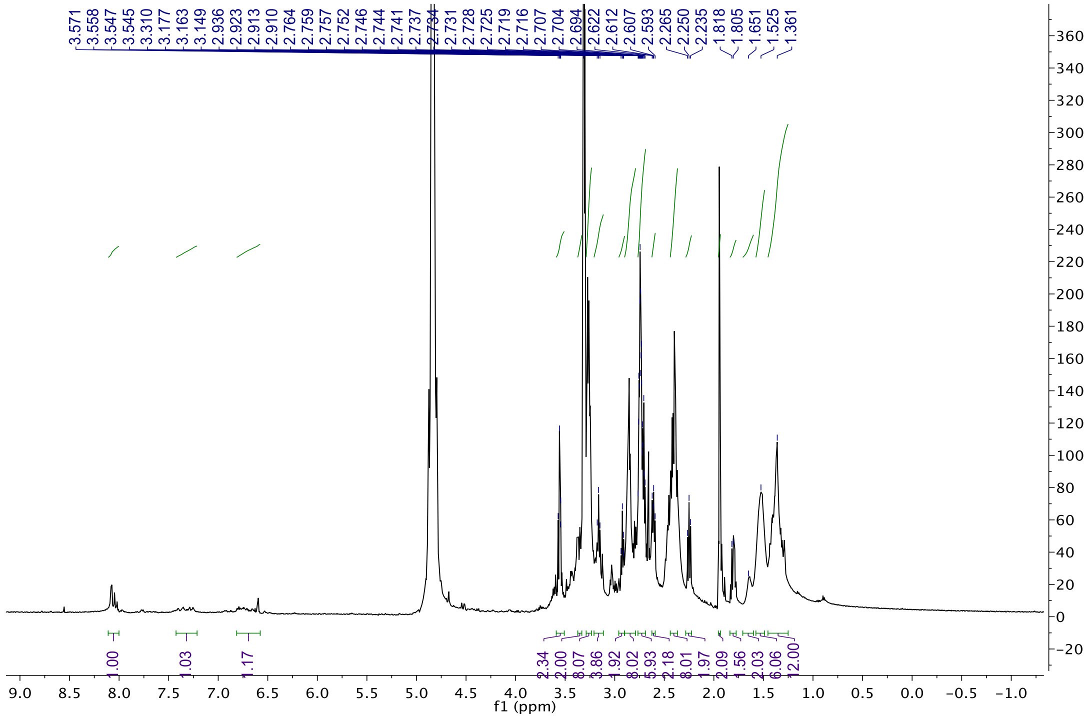

Figure 25. ^{1}H NMR spectrum of compound **4**.

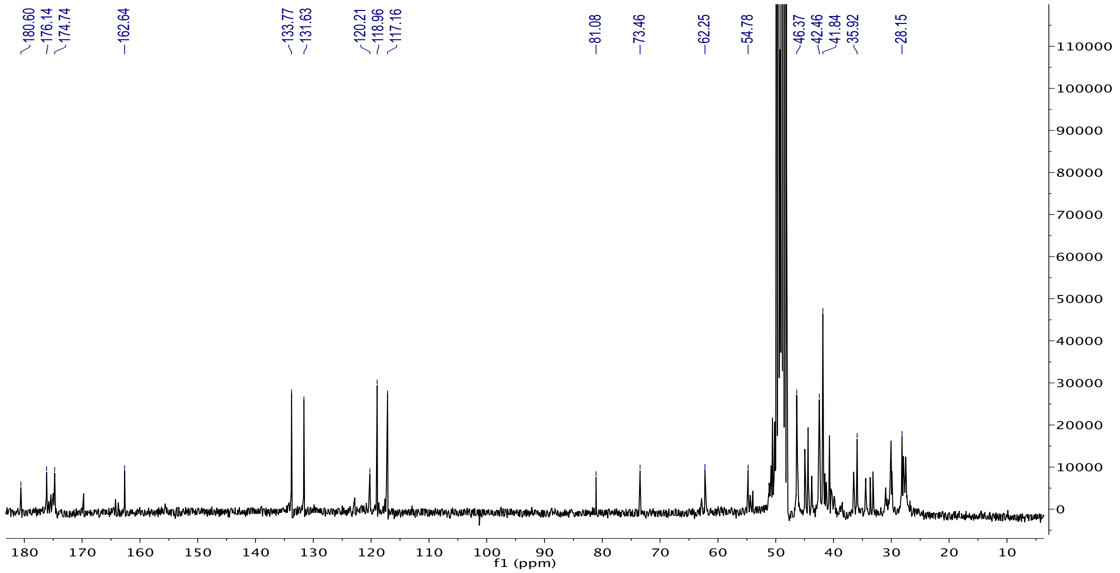

Figure 26. ^{13}C NMR spectrum of compound **4**.

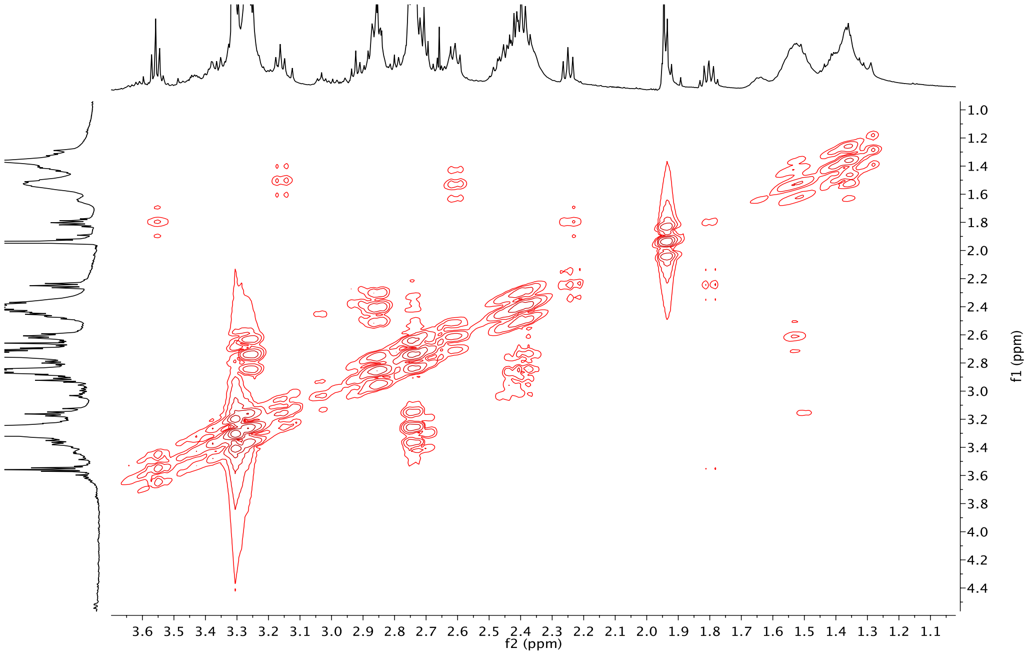

Figure 27. COSY NMR spectrum of compound **4** in the aliphatic region.

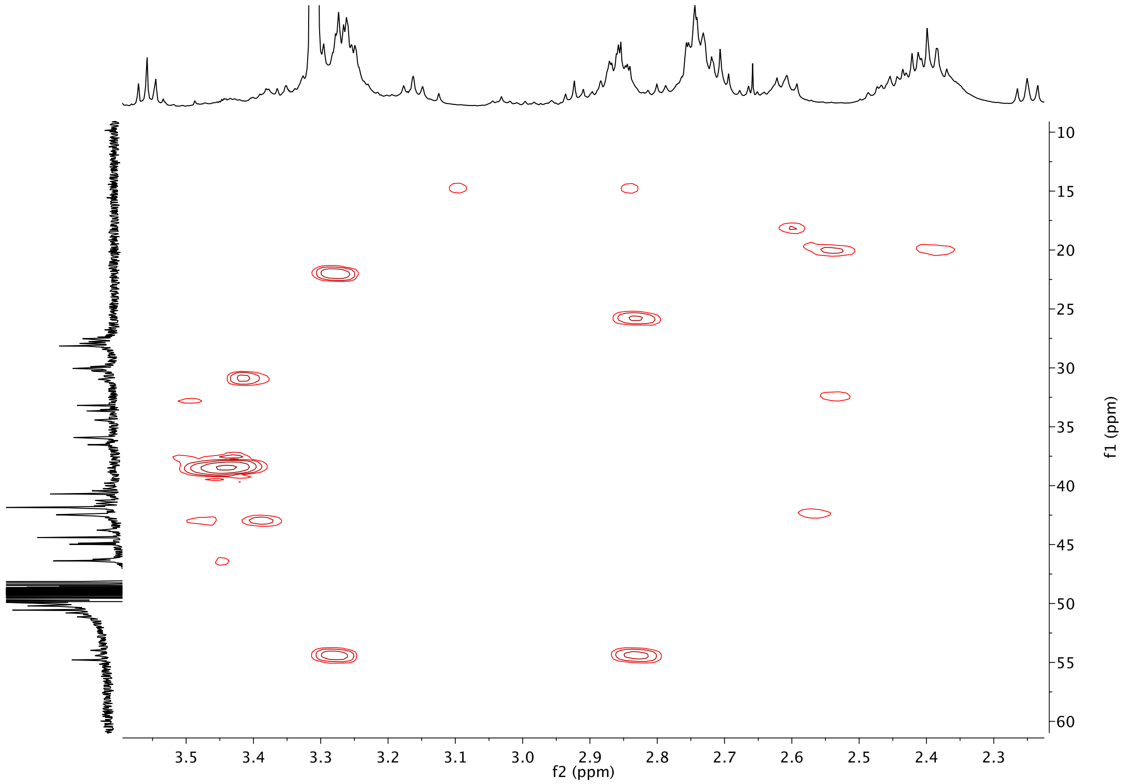

Figure 28. HMBC NMR spectrum of compound **4** in the aliphatic region.

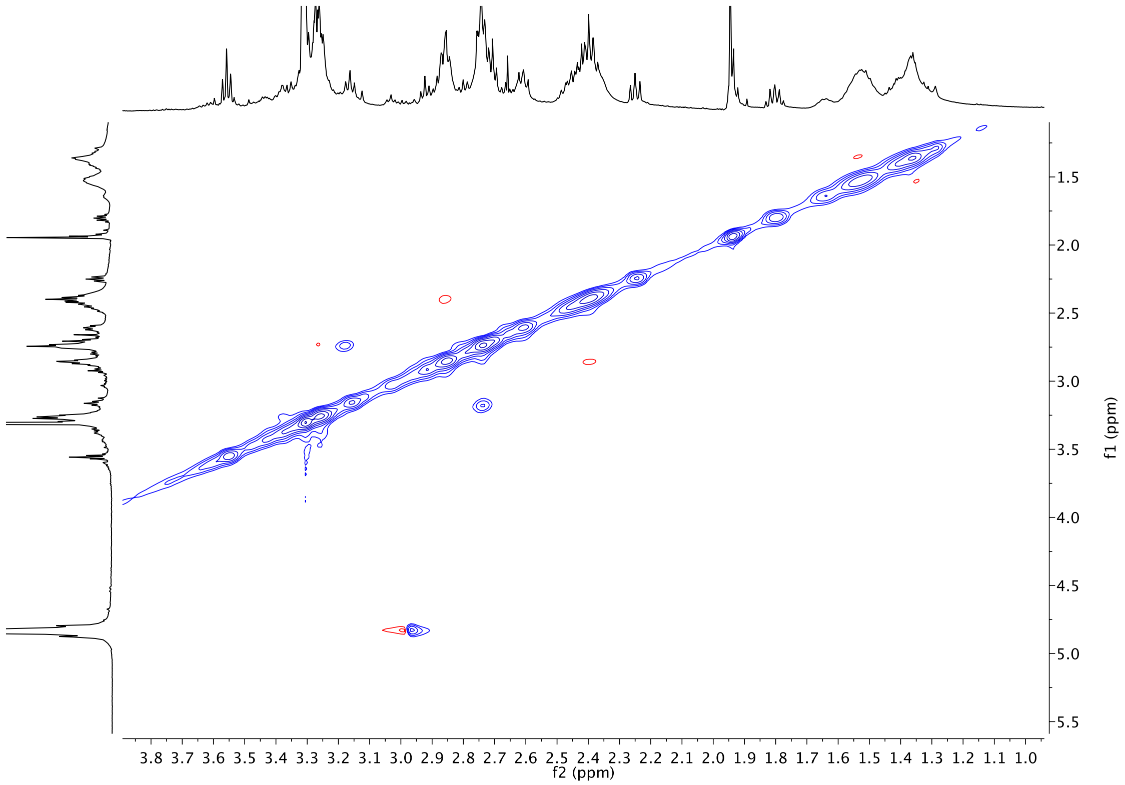

Figure 29. NOESY NMR spectrum of compound **4** in the aliphatic region.

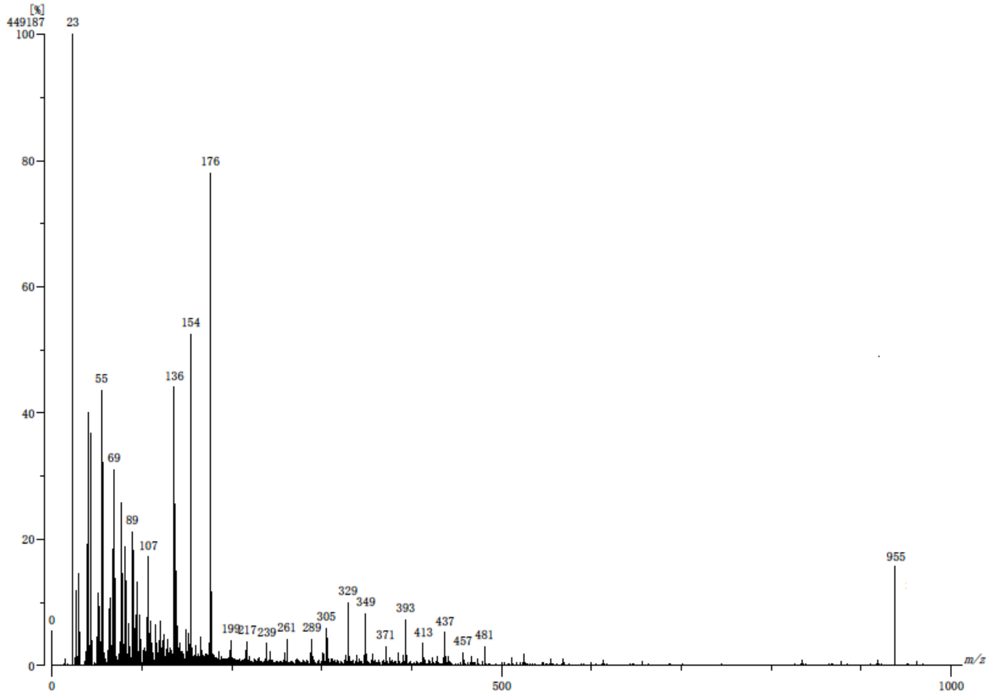

Figure 30. FAB+ mass spectrum of compound **4.**

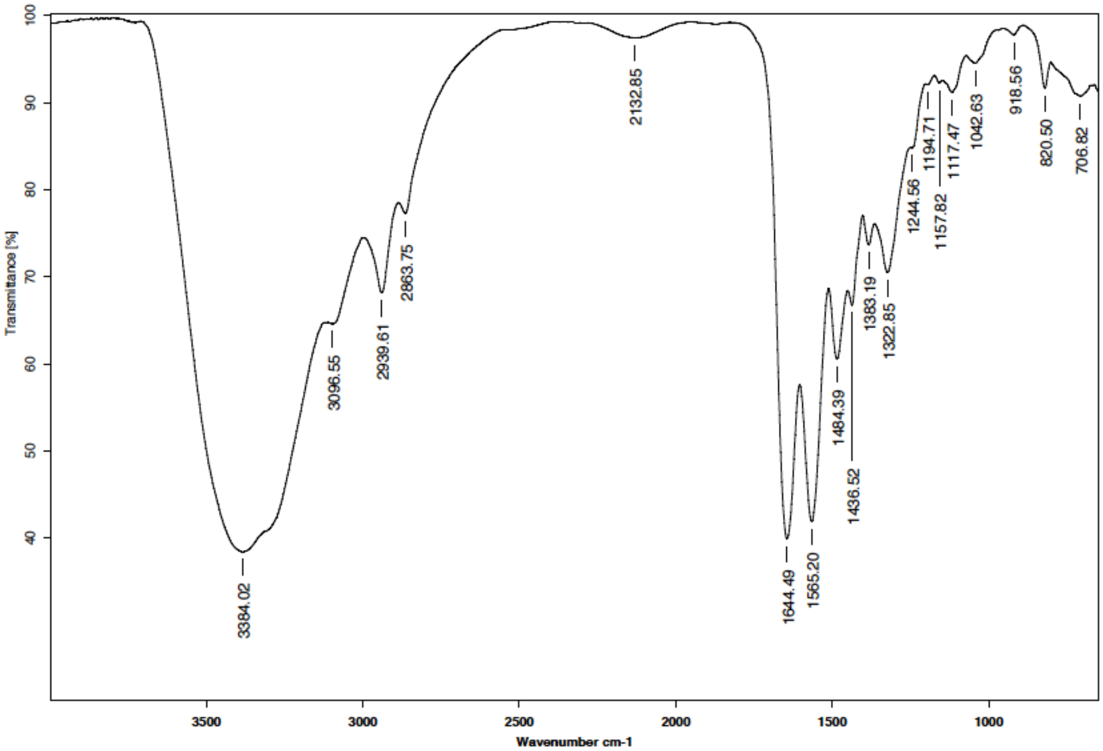

Figure 31. FTIR spectrum of compound **4.**

Compound 6.

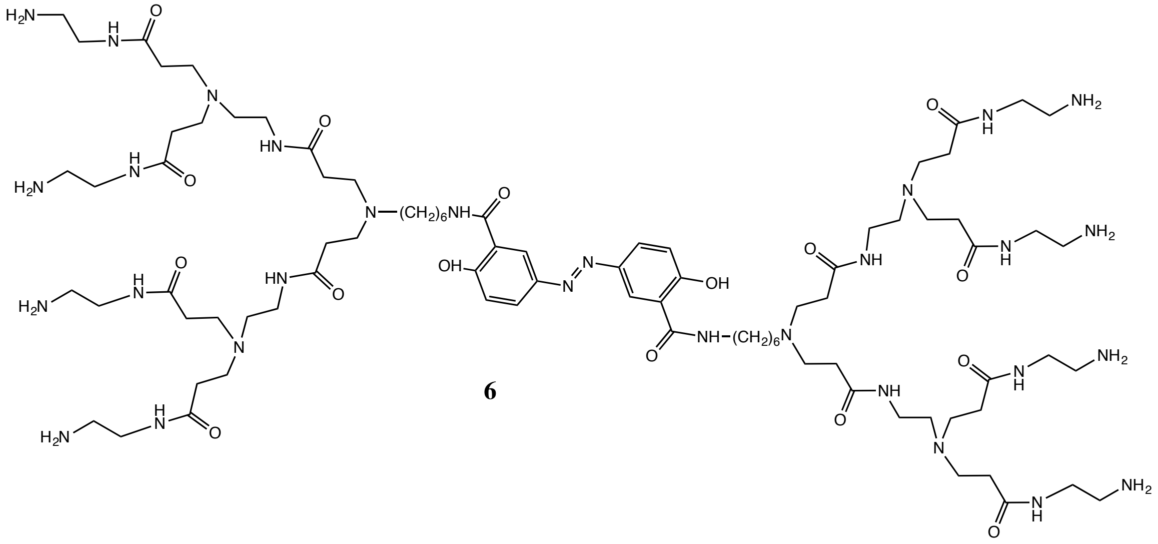

6

Compound **5** (0.439 g, 0.267 mmol) ethylenediamine (0.7 mL, 10.5 mmol). Red liquid, Yield (0.478 g, 0.256 mmol, 96%). UV-Vis (MeOH): $\lambda_{(nm)}$ = 359, 219. IR (Film): $\nu_{(cm^{-1})}$= 3281, 3071, 2928, 2853, 1636. ^{1}H NMR (300 MHz, CD_3OD): $\delta_{(ppm)}$= 1.36 (br, 6H, CH_2),

1.49 (br, 6H, CH_2) 1.80 (t, 2H, J= 6.9 Hz, CH_2), 1.94 (br, 2H, CH_2), 2.12 (m, 2H, CH_2), 2.36 (m, 24H, CH_2), 2.58 (br, 8H, CH_2), 2.72 (br, 24H, CH_2), 2.85 (br, 16H, CH_2), 3.25 (m, 18H, CH_2), 3.38 (br, 4H, CH_2), 3.56 (br, 1H, CH_2), 7.17–7.56 (m, Ar-3), 7.62–7.85 (m, Ar-6), 8.03–8.37 (m, Ar-4). ^{13}C NMR (75 MHz, CD_3OD): $\delta_{(ppm)}$= 27.8 (CH_2), 28.2 (CH_2), 29.9 (CH_2), 30.3 (CH_2), 33.2 (CH_2-*N*), 33.7 (CH_2-*N*), 34.7 (CH_2-*NH*), 36.5 (CH_2-*NH*), 40.7 (CH_2-*C=O*), 41.9 (CH_2-*C=O*), 42.8 (CH_2-*NH2*), 44.4 (CH_2-*N*), 45.0 (CH_2-*NH*), 46.2 (CH_2-*NH*), 50.6 (CH_2-*N*), 51.1 (CH_2-*N*), 169.7 (C=O), 175.3 (C=O), 180.6 (C=O). EM FAB+): m/z = 1867 $[M]^+$. Elemen. Anal. calc. for. $C_{86}H_{158}N_{30}O_{16}$. C 55.28, H 8.52, N 22.49, O, 13.70 %. Found: C 55.27, H 8.51, N 22.50 %.

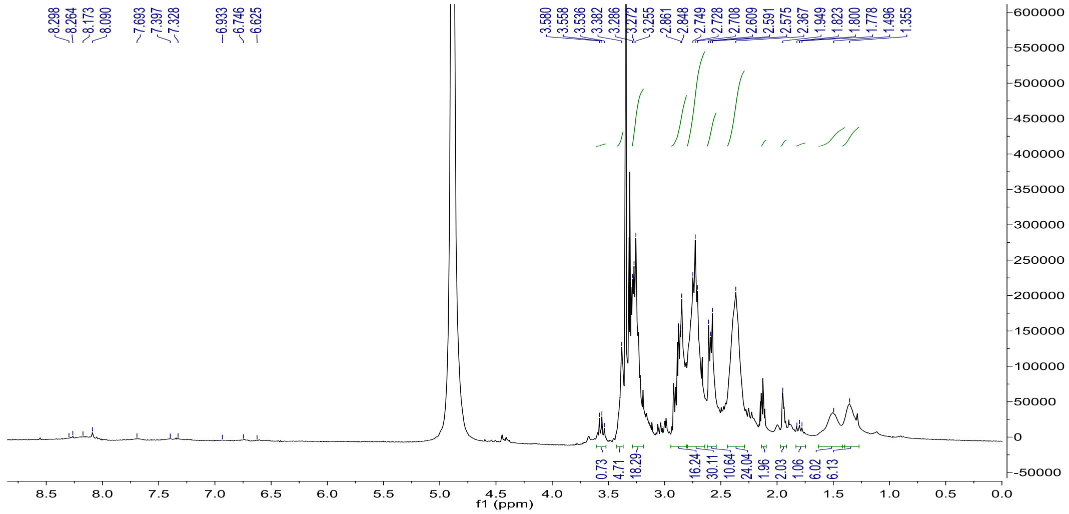

Figure 32. ^{1}H NMR spectrum of compound **6**.

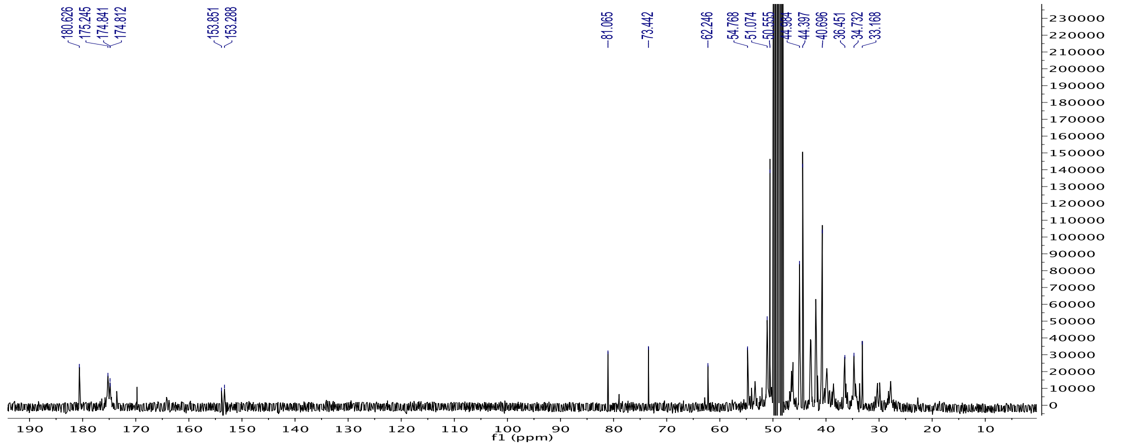

Figure 33. ^{13}C NMR spectrum of compound **6**.

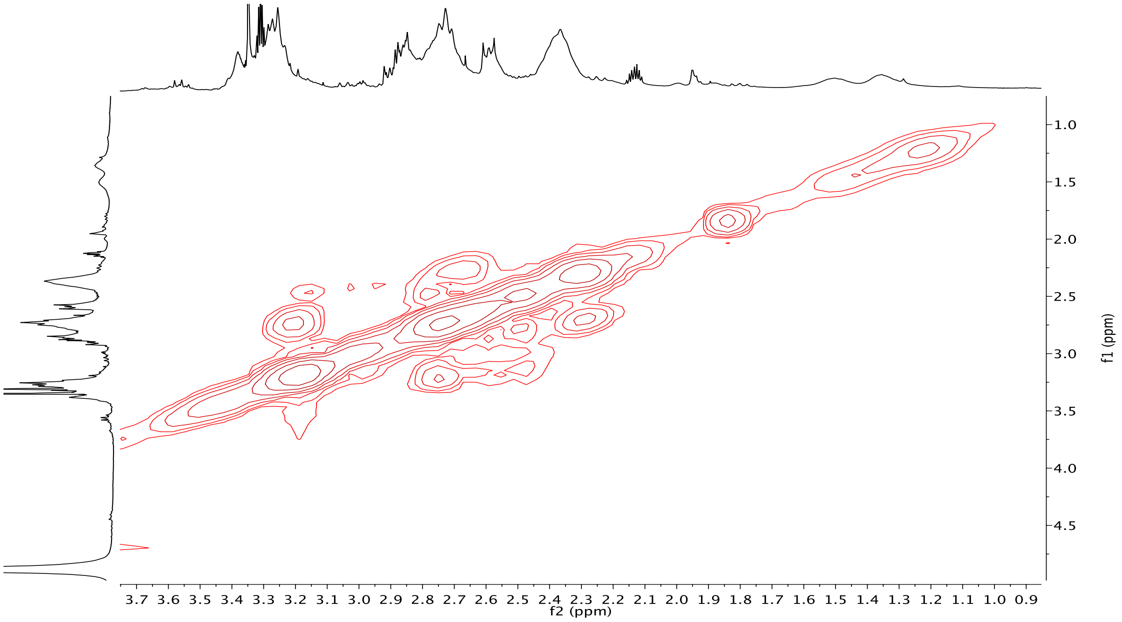

Figure 34. COSY NMR spectrum of compound **6** in the aliphatic region.

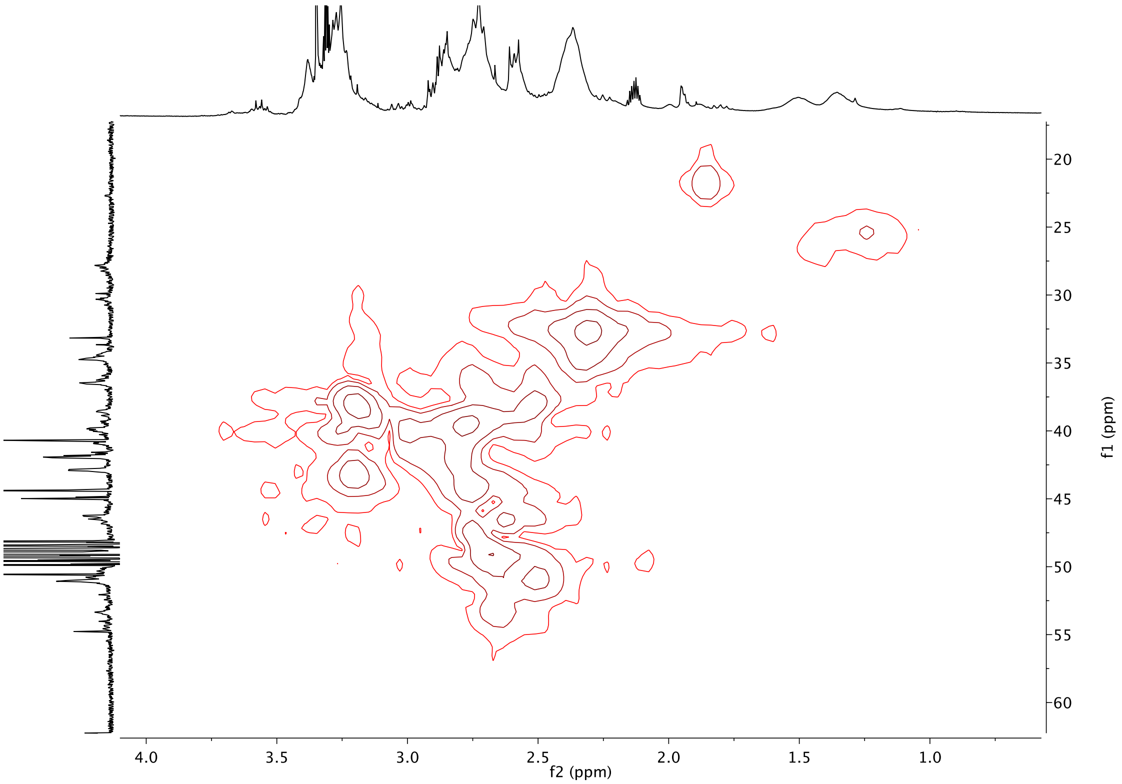

Figure 35. HMQC NMR spectrum of compound **6** in the aliphatic region.

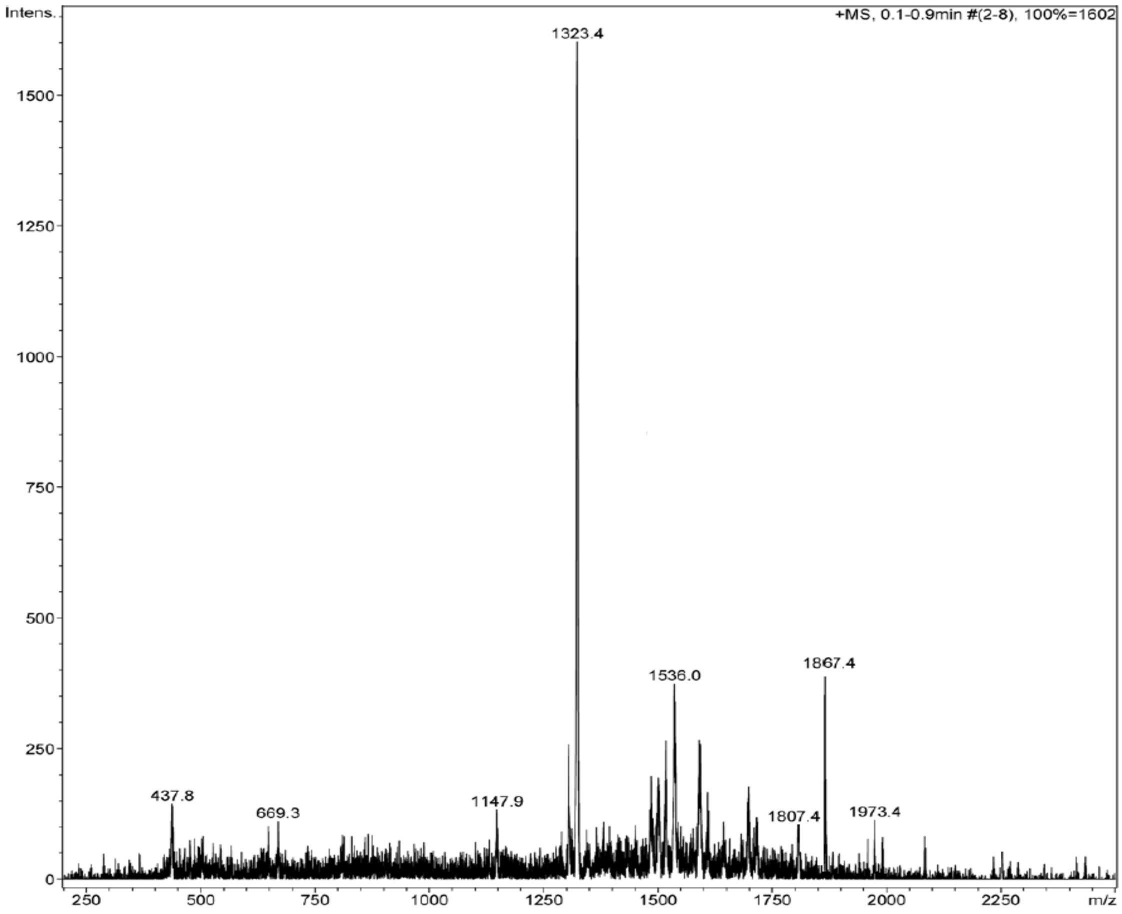

Figure 36. ESI mass spectrum of compound **6.**

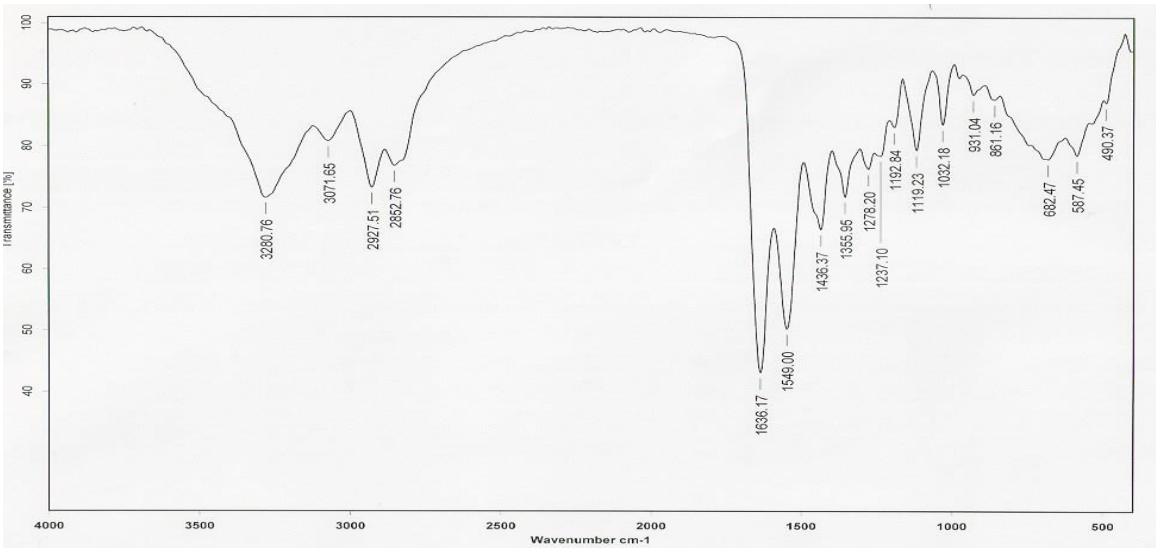

Figure 37. FTIR spectrum of compound **6.**

Synthesis of the methyl salicylate 7.

To a solution of salicylic acid (5.0 g, 36.20 mmol) in methanol, H_2SO_4 (4.5 mL) was slowly added, the mixture was refluxed for 24 h. After that, 100 mL of water and CH_2Cl_2 were added, the organic phase was separated and washed with $NaHCO_3$, the mixture was filtered over Na_2SO_4 and the organic phase was evaporated under vacuum obtaining the compound 7 (3.490 g, 22.94 mmol, 63%) of yellow color.

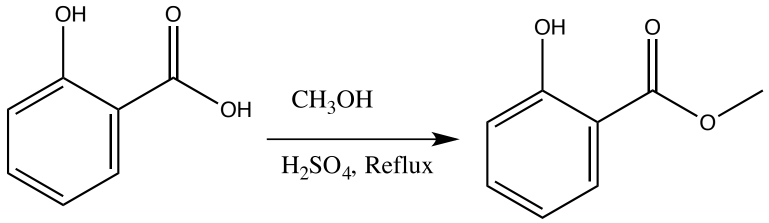

7

Scheme 5. Synthesis of the methyl salicylate **7**.

UV-Vis (CH_2Cl_2): $\lambda_{(nm)}$= 304, 232. IR (film): $\nu_{(cm^{-1})}$= 3188, 2956, 2854, 1679. ^{1}H NMR (300 MHz, $CDCl_3$): $\delta_{(ppm)}$= 3.94 (s, 3H, CH_3), 6.87 (ddd, 1H, *J*= 8.2, 7.3, 1.1 Hz, Ar-5), 6.97 (dd, 1H, *J*= 8.4, 0.8 Hz, Ar-3), 7.44 (ddd, 1H, *J*= 8.6, 7.3, 1.6 Hz, Ar-4), 7.82 (dd, 1H, *J*= 8.0, 1.5 Hz, Ar-6), 10.75 (s, 1H, OH). ^{13}C NMR (300 MHz, $CDCl_3$): $\delta_{(ppm)}$= 52.4 (CH_3), 112.5 (Ar-1), 117.6 (Ar-3), 119.3 (Ar-5), 130.0 (Ar-6), 135.8 (Ar-4), 161.7 (Ar-2), 170.7 (C=O). EM (DART): m/z =153 $[M+1]^+$. Elemen. Anal. calc. for. $C_8H_8O_3$. C 63.15, H 5.30, O 13.70 %. Found: C 63.17, H 5.30 %.

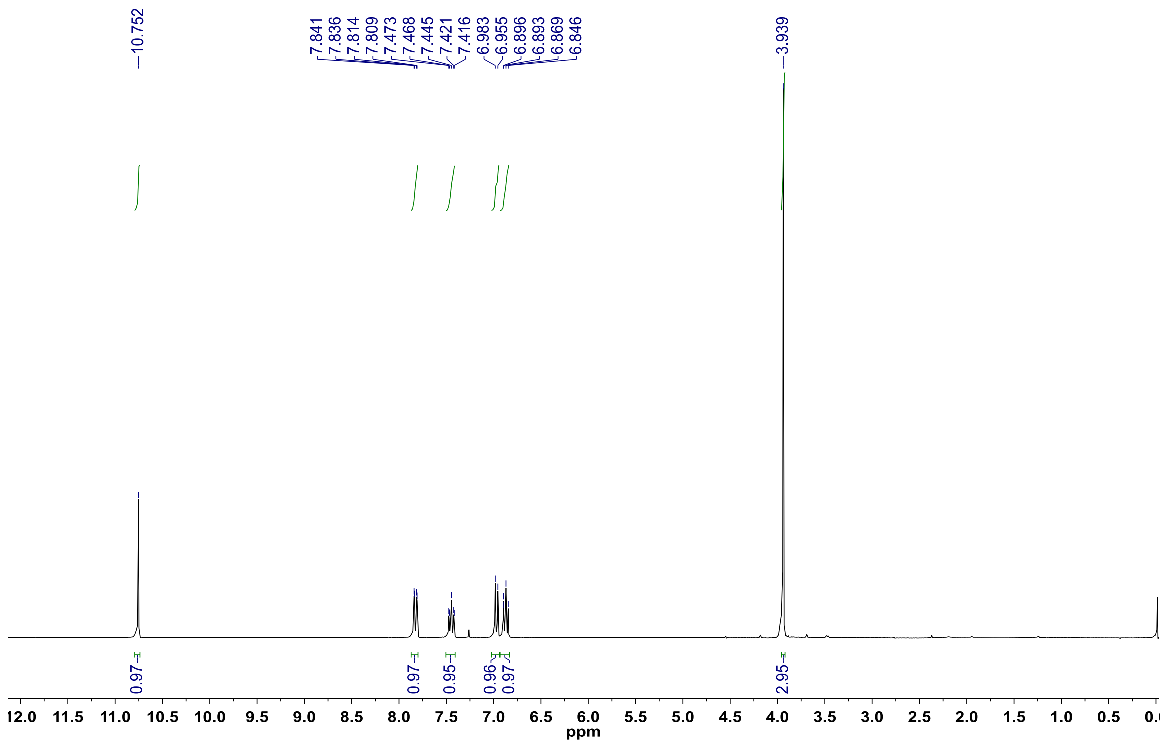

Figure 38. ^{1}H NMR spectrum of compound **7**.

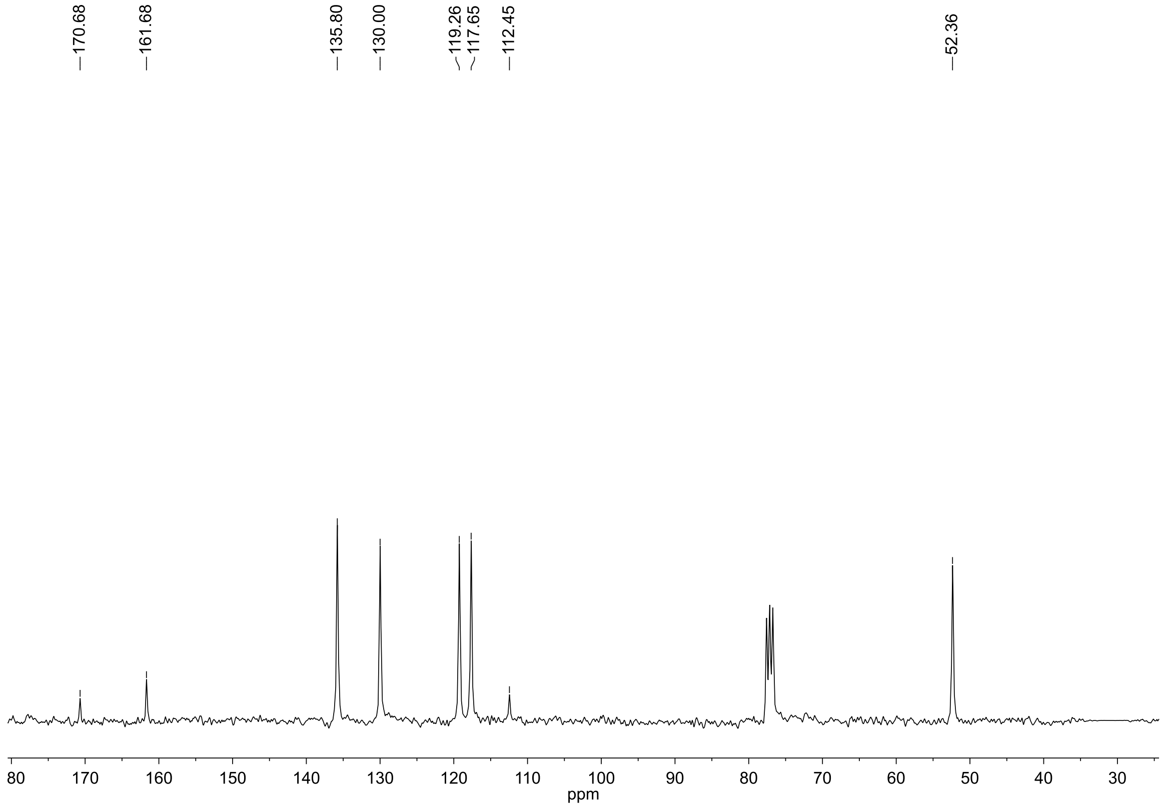

Figure 39. ^{13}C NMR spectrum of compound **7**.

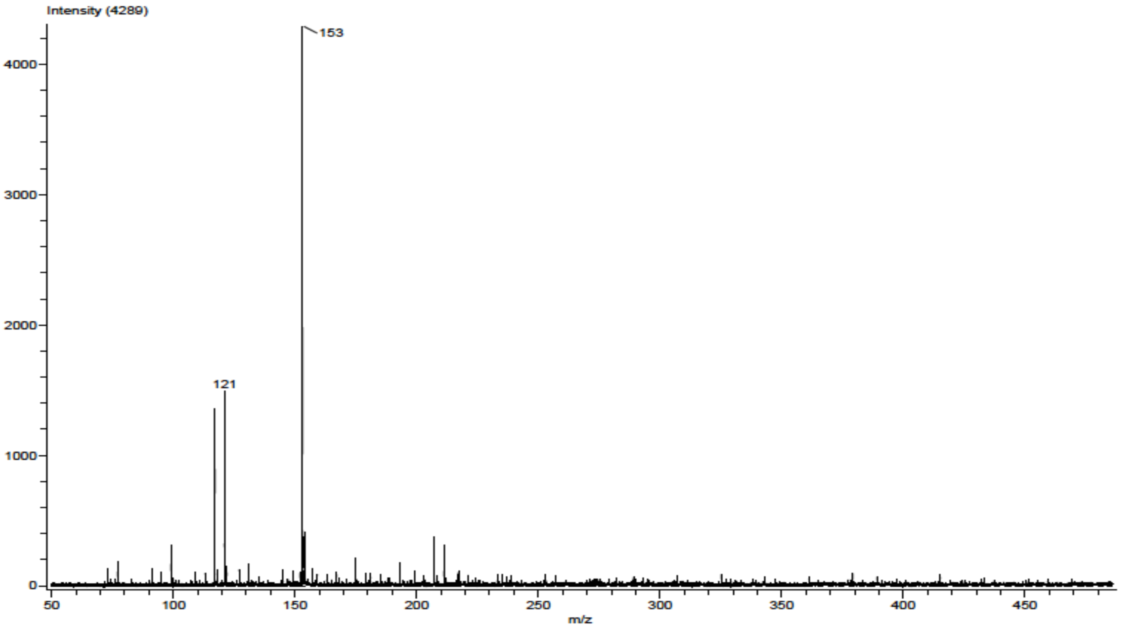

Figure 40. Mass spectrum of compound **7**.

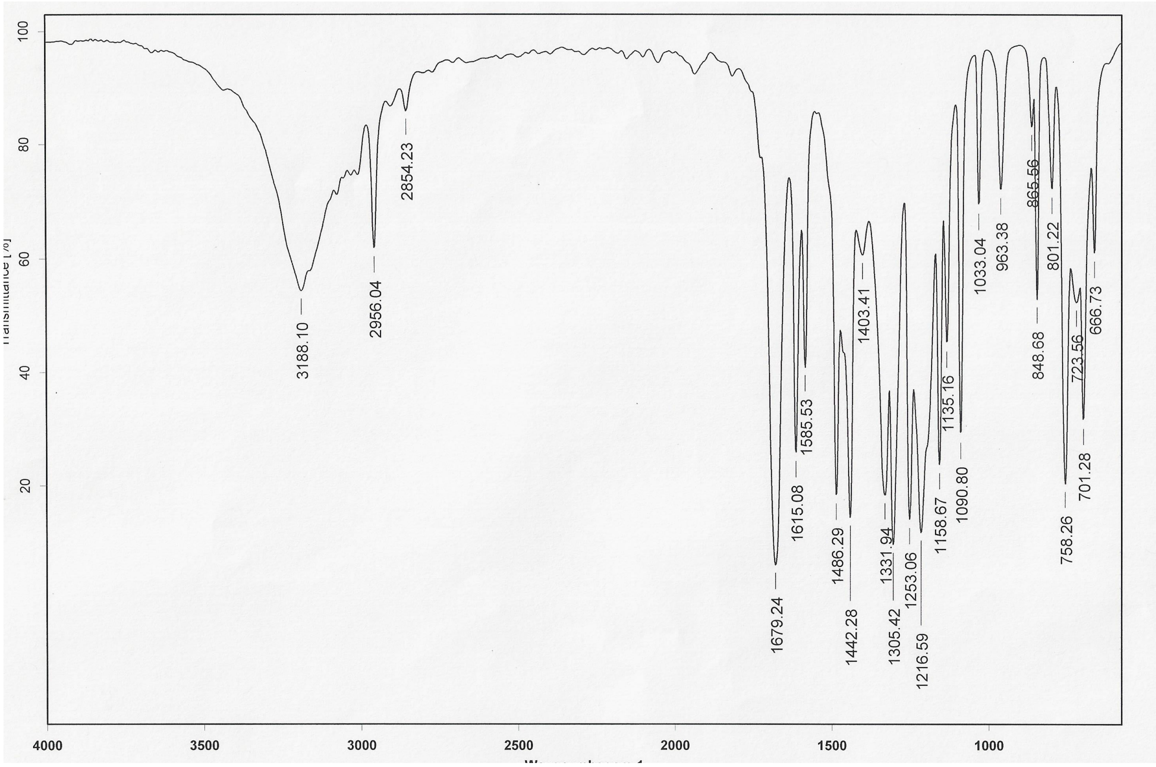

Figure 41. FTIR spectrum of compound **7**.

Synthesis of the conjugates 8 and 9.

Compounds **4** or **6** (0.045 mmol) were dissolved in methanol:toluene 5:1 (40 mL) and heated to 80°C. After 20 min, methyl salicylate (1.84 mmol) was added in methanol. The mixture was stirred and heated to 120 °C for 3 d. The solvent was evaporated and the resulting solid was washed with hexane giving the conjugates **8** or **9**, respectively.

Compound 8.

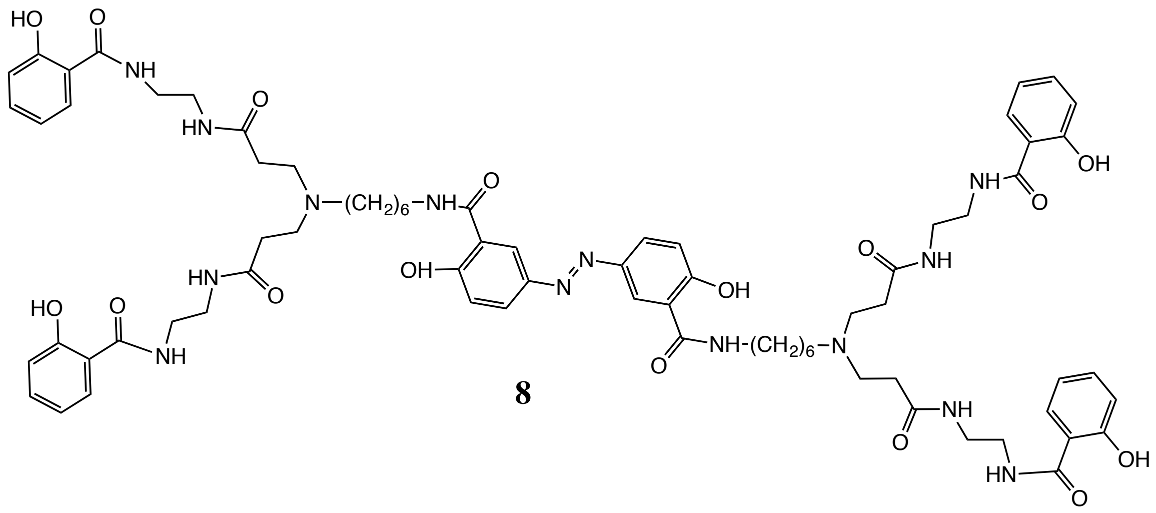

8

Red paste. Yield 0.513 g, 0.45 mmol, 80%. UV-Vis (MeOH): $\lambda_{(nm)}$= 302, 215. IR (Película): $\nu_{(cm^{-1})}$= 3263, 3068, 2929, 2858, 1621. ^{1}H NMR (300 MHz, CD_3OD): $\delta_{(ppm)}$= 1.22–1.44 (br, 10H, CH_2), 1.45–1.72 (br, 2H, CH_2), 1.73–1.94 (br, 2H, CH_2), 2.10–2.44 (br, 4H, CH_2-C=O), 2.44–2.61 (br, 1H, NH), 2.62 – 2.67 (br, 4H, NH), 2.67 – 2.78 (br, 1H, CH_2), 3.34–3.46 (br, 7H, CH_2-NH), 3.52 (br, 6H, CH_2-NH), 3.62 (br, 14H, CH_2-NH), 3.90 (br, 6H, CH_2), 6.72–7.18 (br, 16H, Ar), 7.22–7.52 (br, 3H, Ar), 7.73–8.02 (br, 3H, Ar). ^{13}C NMR (75 MHz, CD_3OD): $\delta_{(ppm)}$= 27.0 (CH_2), 27.5 (CH_2), 30.2 (CH_2), 30.3 (CH_2), 33.7 (CH_2-N), 40.0 (CH_2-NH), 40.3 (CH_2-C=O), 40.7 (CH_2-NH), 45.0 (CH_2-NH), 52.4 (CH_2-N), 117.1 (Ar), 119.0 (Ar), 119.6 (Ar), 129.0 (Ar), 131.6 (Ar), 133.8 (Ar), 134.7 (Ar), 162.0 (Ar), 162.6 (Ar), 171.6 (C=O), 176.1 (C=O). EM (ESI): m/z =1434 $[M+1]^{+}$. Elemen. Anal. calc. for. $C_{74}H_{94}N_{14}O_{16}$: C, 61.91; H, 6.60; N, 13.66, O 17.83 %. Found: C 61.93, H 6.57, N 13.66 %.

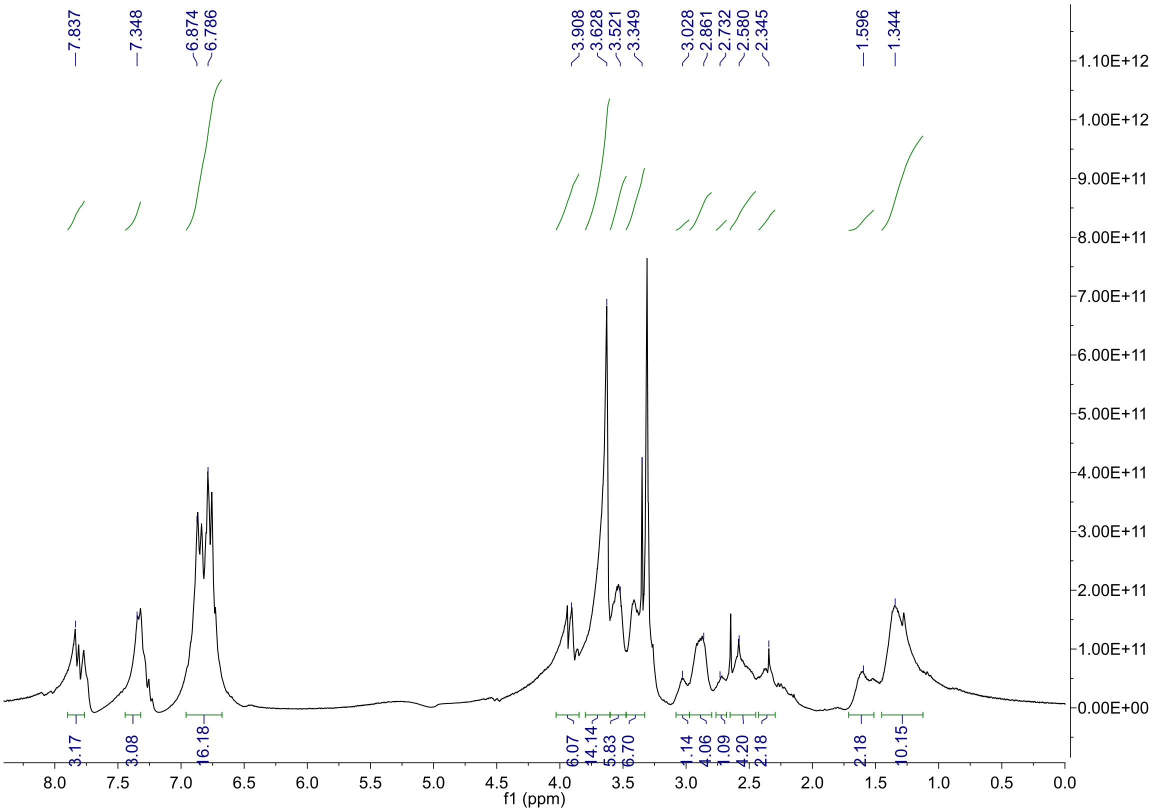

Figure 42. ^{1}H NMR spectrum of compound **8**.

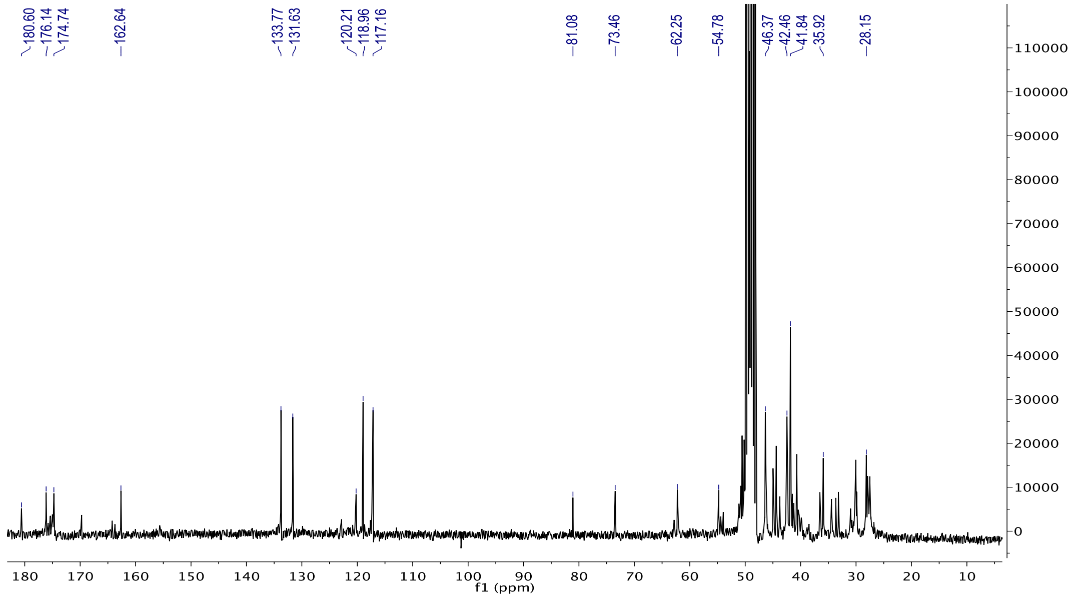

Figure 43. ^{13}C NMR spectrum of compound **8**.

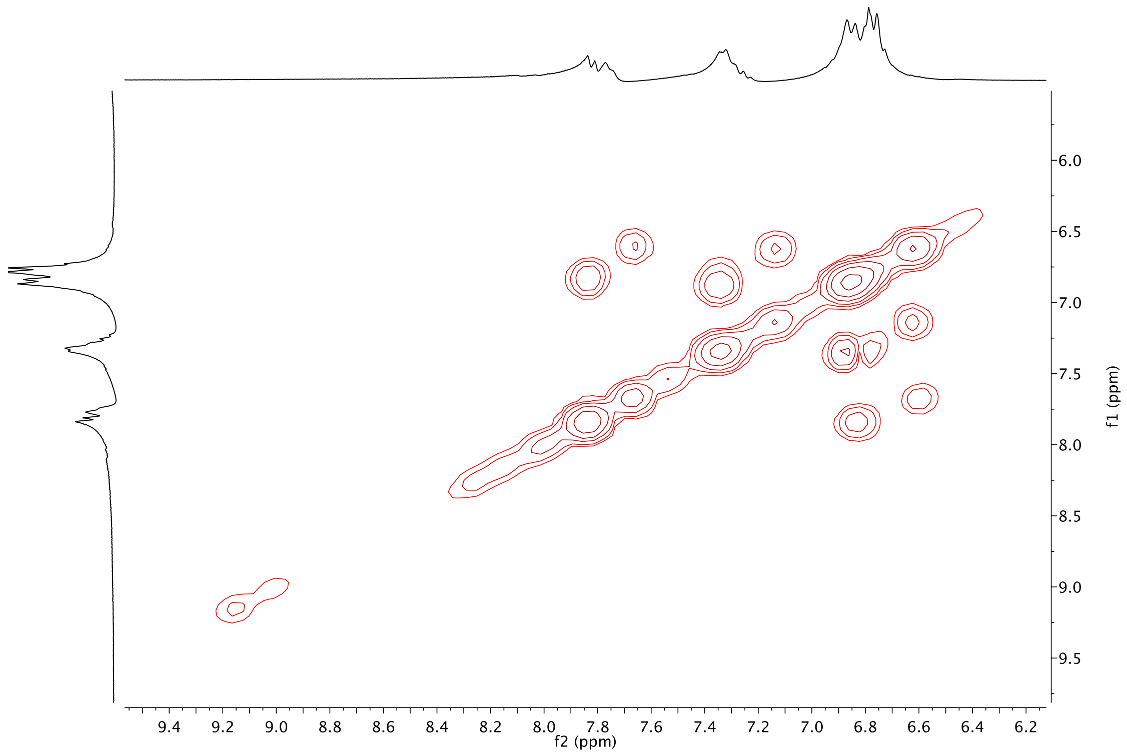

Figure 44. COSY NMR spectrum of compound **8** in the aromatic region.

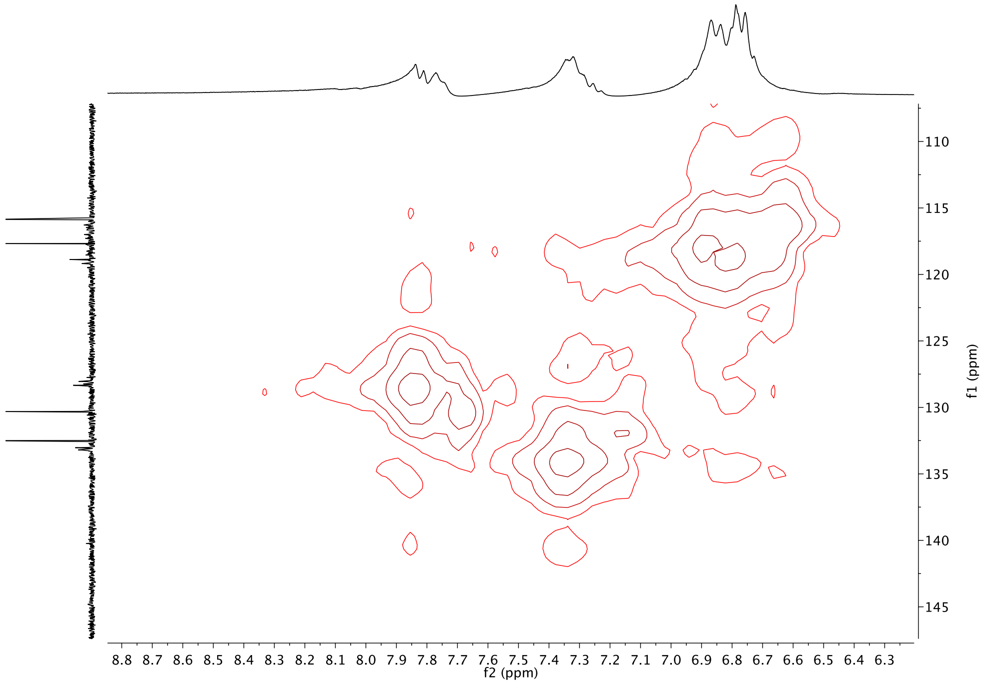

Figure 45. HMQC NMR spectrum of compound **8** in the aromatic region.

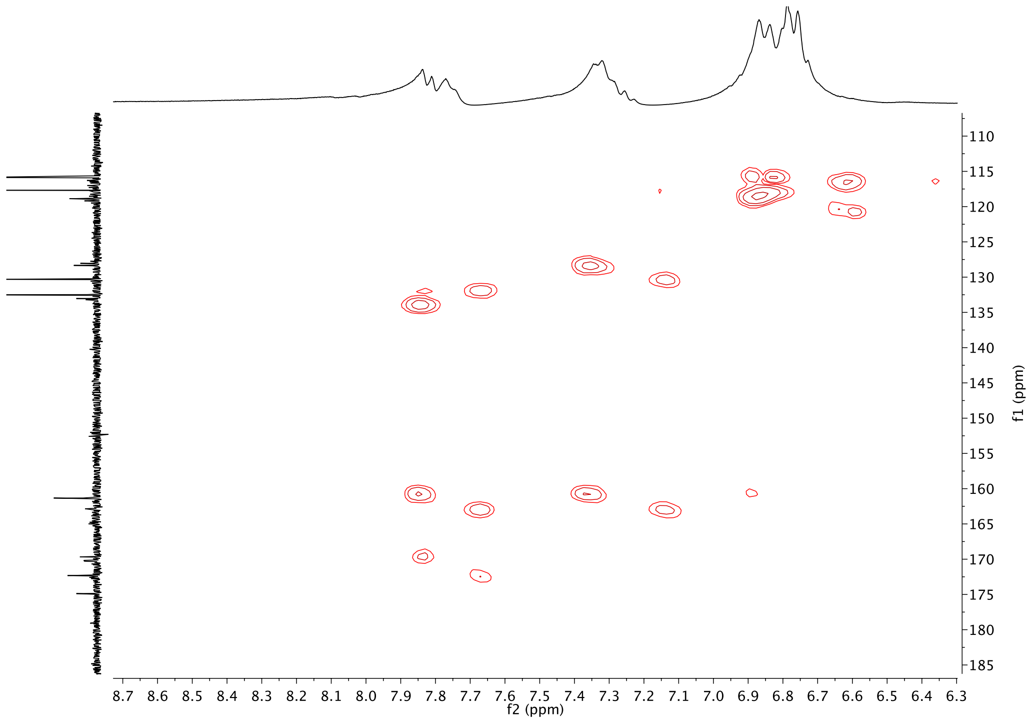

Figure 46. HMBC NMR spectrum of compound **8** in the aromatic region.

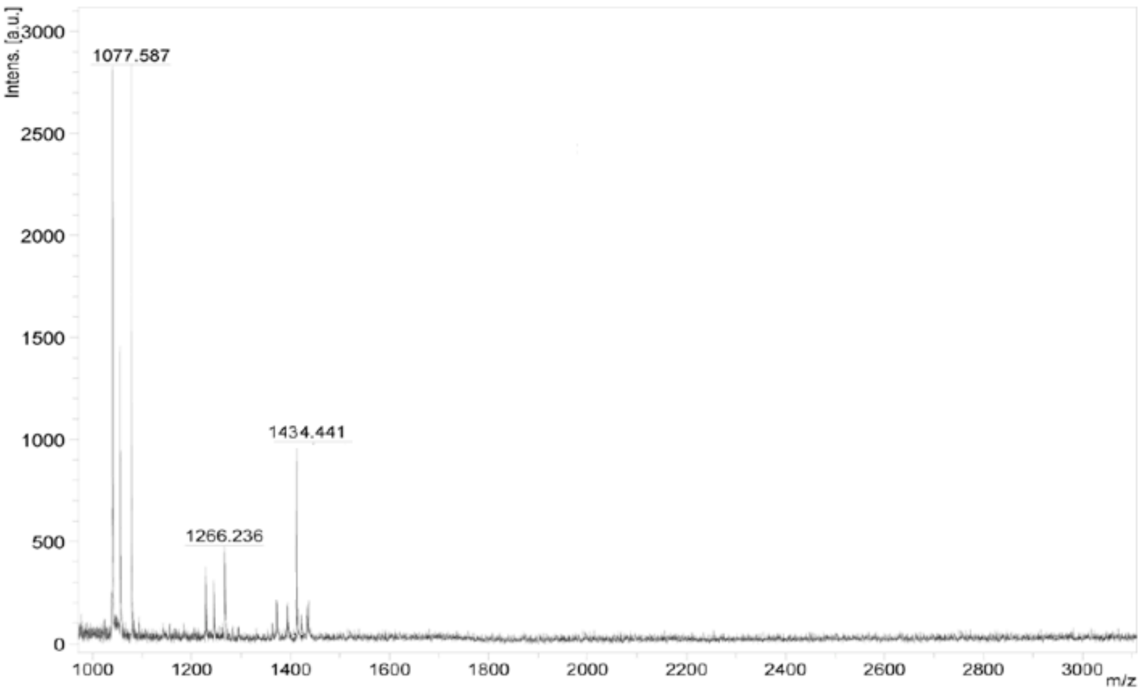

Figure 47. MALDI-TOF Mass spectrum of compound **8**.

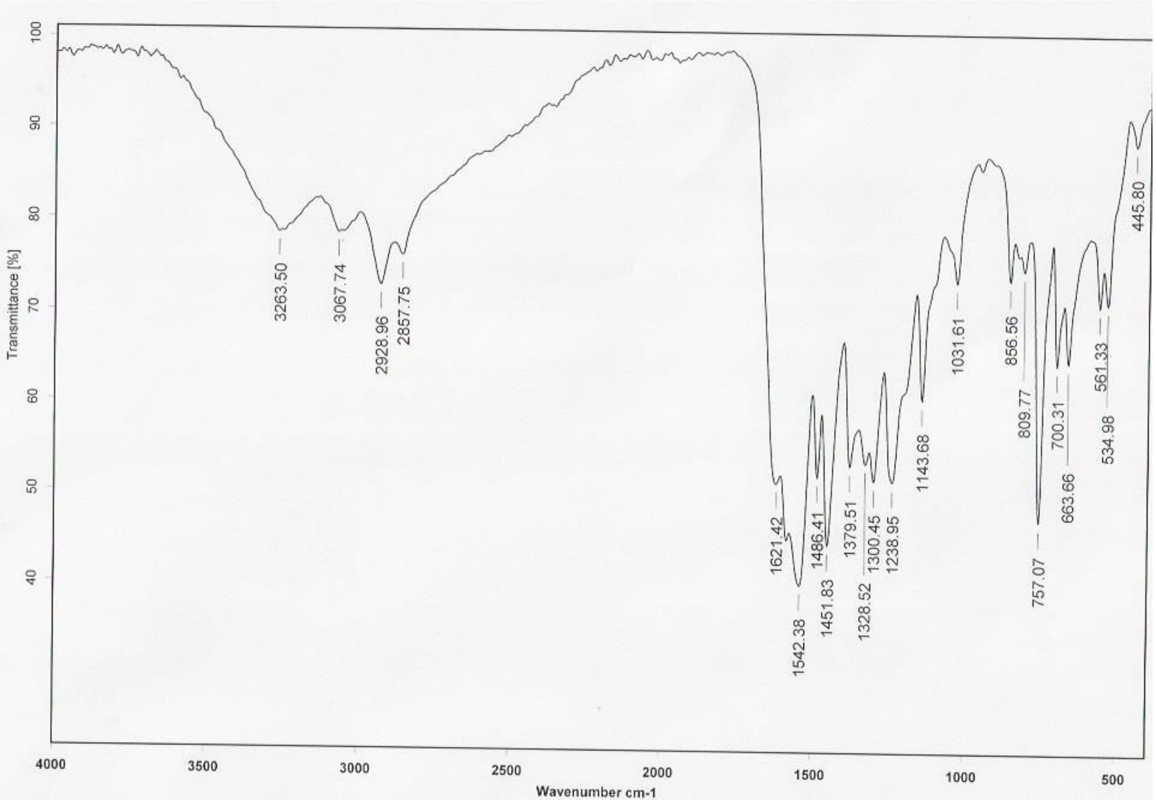

Figure 48. FTIR spectrum of compound **8**.

Compound 9.

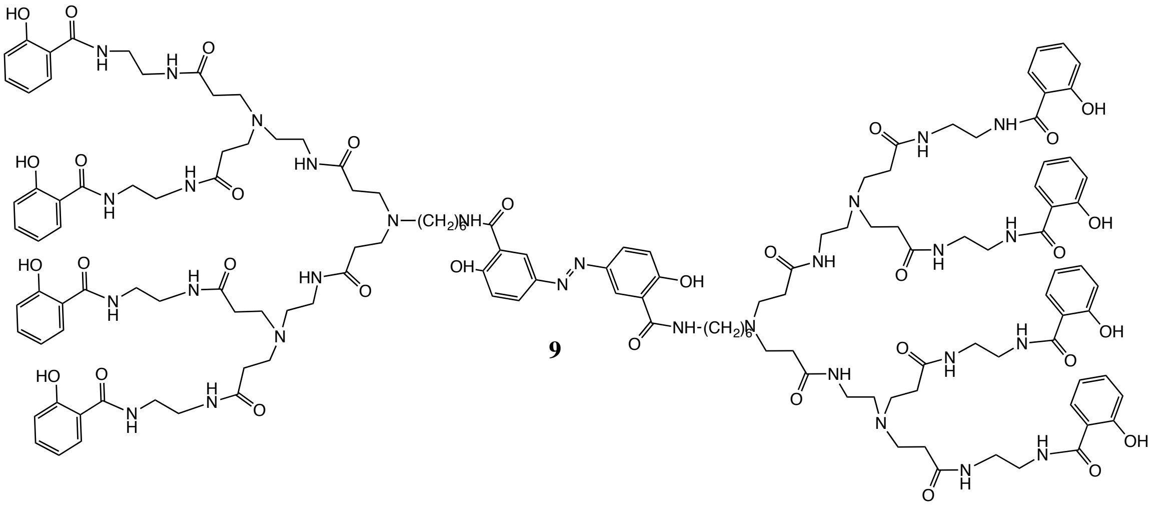

9

Brown paste. (0.303 g, 0.11 mmol, 89%). UV-Vis (MeOH): $\lambda_{(nm)}$ = 300, 206. IR, film $\nu_{(cm^{-1})}$= 3270, 3076, 2932, 2827, 1631, 1588. ^1H NMR (300 MHz, CD_3OD): $\delta_{(ppm)}$= 1.24–1.43 (br, 8H, CH_2), 1.46–1.69 (br, 8H, CH_2), 2.22–2.44 (br, 24H, CH_2-C=O), 2.56–2.65 (br, 8H, CH_2-N, CH_2-NH), 2.70–2.79 (br, 8H, CH_2-N), 2.82–3.04 (br, 24H, CH_2-N), 3.25–3.30 (br, 8H, CH_2-NH), 3.34–3.66 (m, 54H, CH_2-NH, NH), 6.74–7.02 (br, 18H, Ar), 7.21–7.54 (br, 10H, Ar), 7.70–8.02 (br, 10H, Ar). ^{13}C NMR (75 MHz, CD_3OD): $\delta_{(ppm)}$= 27.5 (CH_2), 30.2 (CH_2), 33.2 (CH_2-N), 33.7 (CH_2-N), 34.7 (CH_2-NH), 35.9 (CH_2-NH), 40.0 (CH_2-C=O), 42.9 (CH_2-N), 44.3 (CH_2-NH), 44.8 (CH_2-NH), 46.1 (CH_2-NH), 50.5 (CH_2-N), 51.2 (CH_2-N), 117.2 (Ar), 119.0 (Ar), 119.5 (Ar), 129.0 (Ar), 131.6 (Ar), 133.8 (Ar), 134.7 (Ar), 162.2 (Ar), 171.4 (C=O), 176.1 (C=O). EM (ESI): m/z = 2828 $[M+1]^+$. Elemen. Anal. calc. for. $C_{142}H_{190}N_{30}O_{32}$:. C 60.28, H 6.77, N 14.85, O 18.10 %. Found: C 60.27, H 6.74 %.

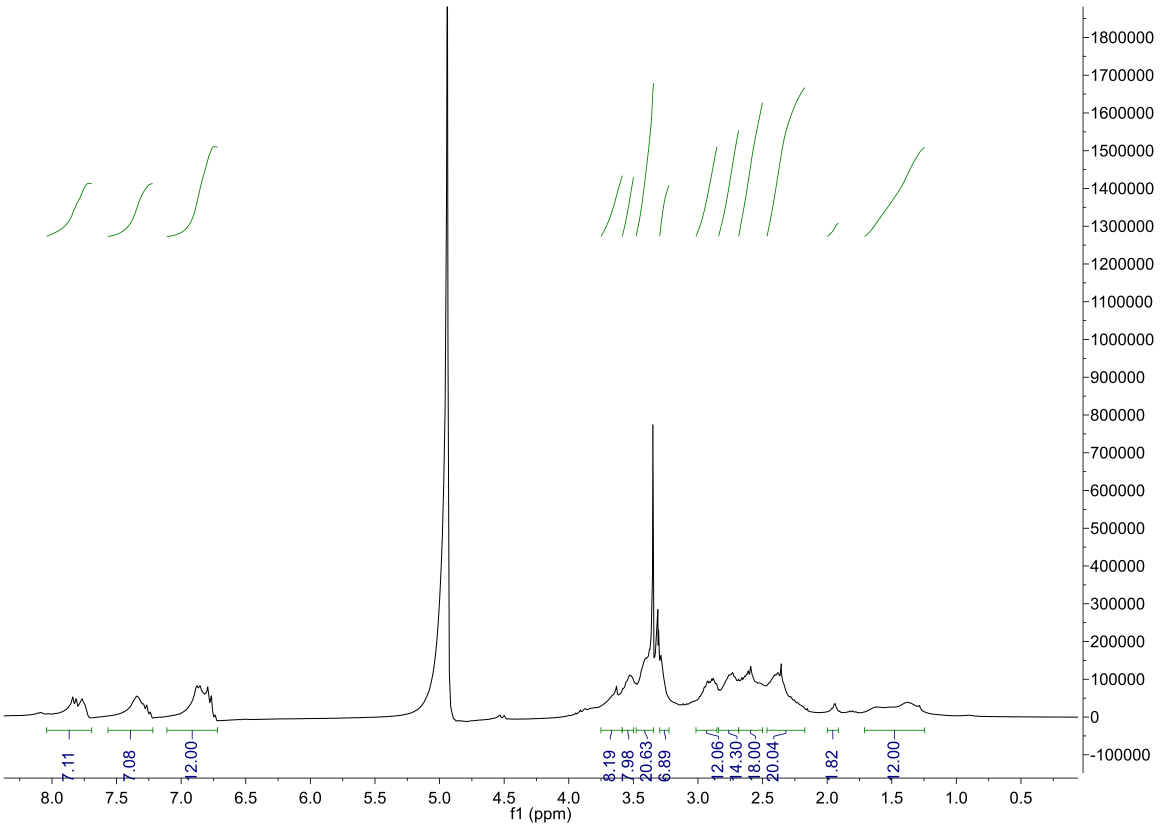

Figure 49. ^1H NMR spectrum of compound **9** in CD_3OD.

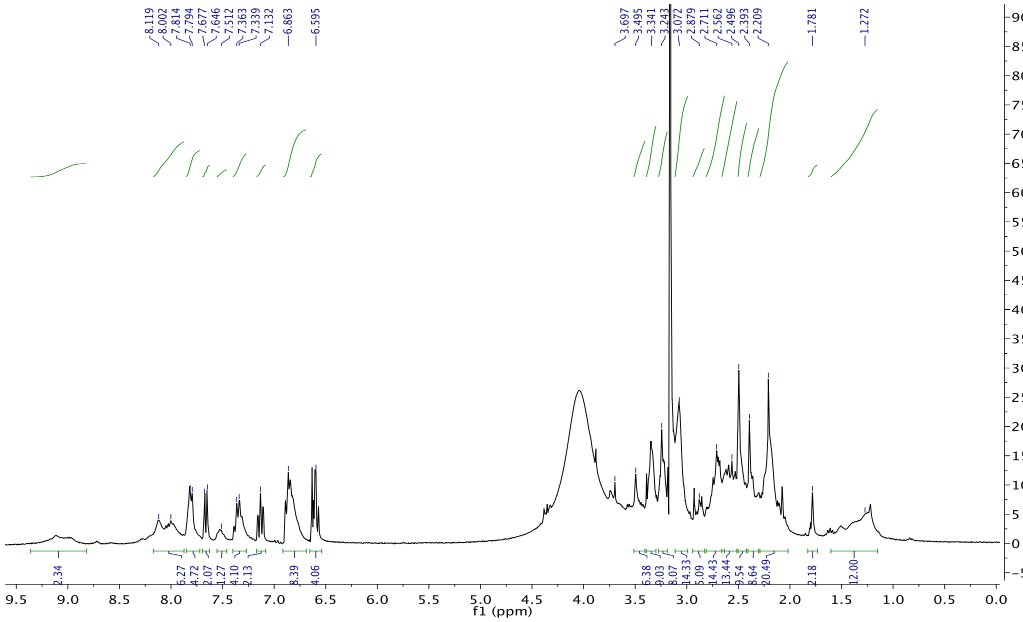

Figure 50. ^{1}H NMR spectrum of compound **9** in DMSO-d_6.

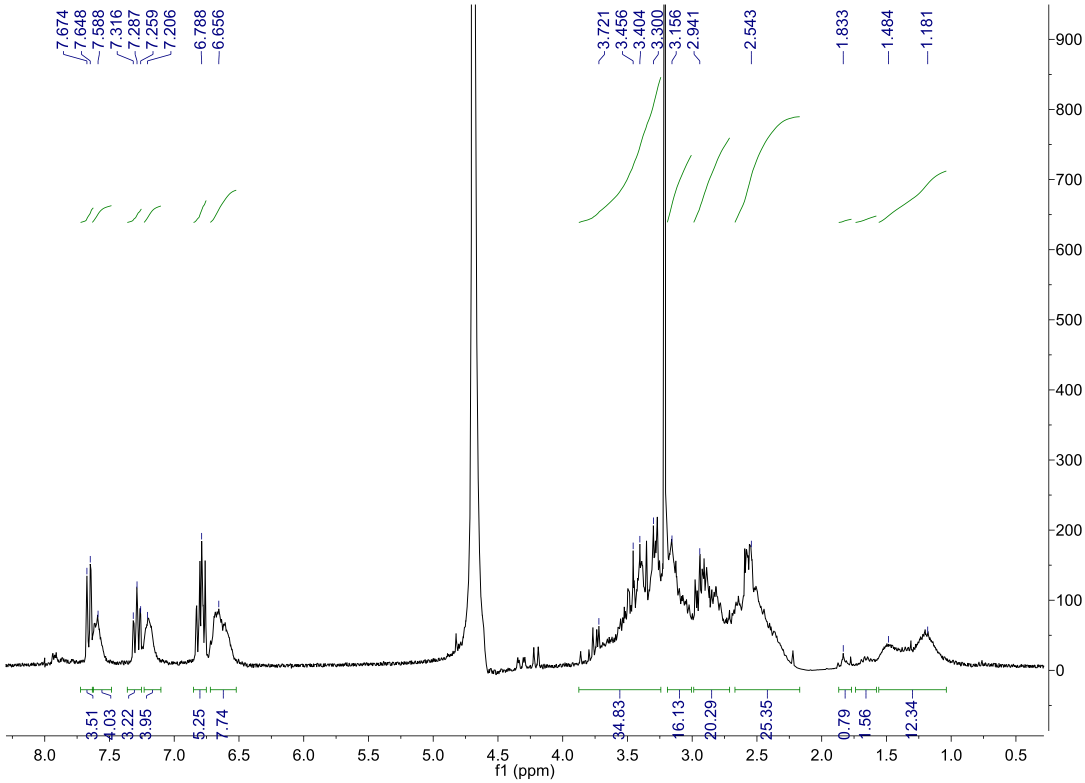

Figure 51. ^{1}H NMR spectrum of compound **9** in D_2O.

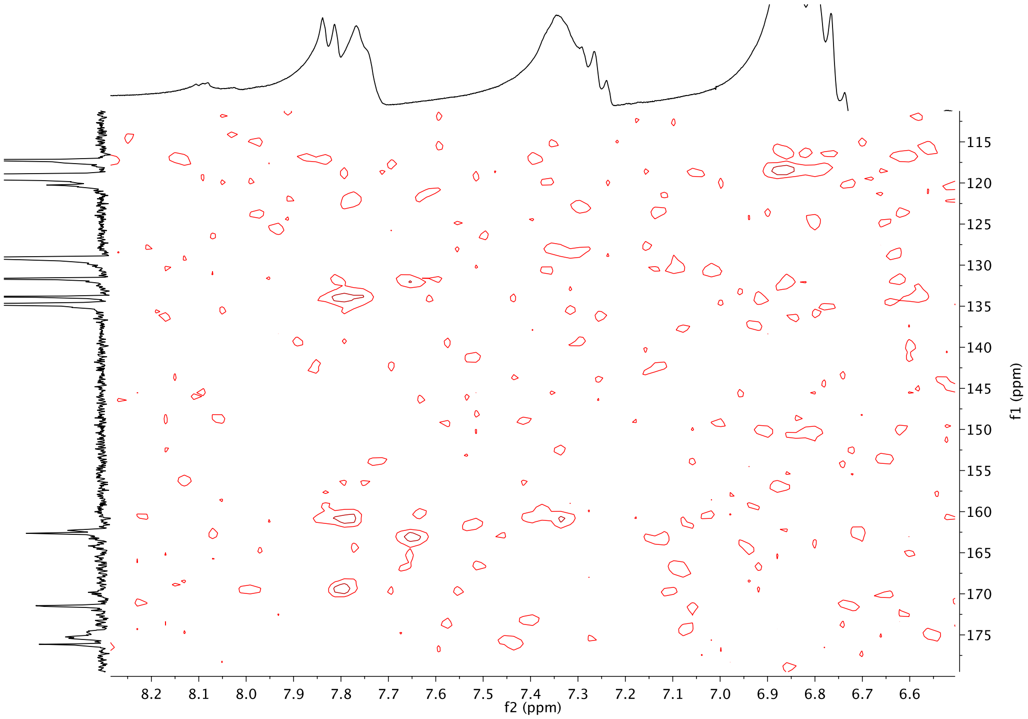

Figure 52. HMBC NMR spectrum of compound **9** in the aromatic region in CD_3OD.

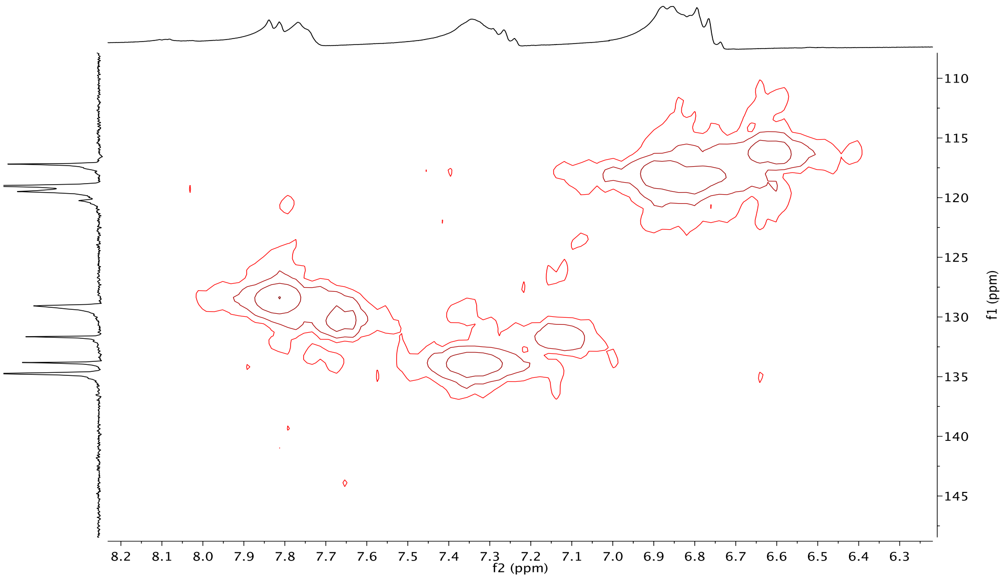

Figure 53. HMQC NMR spectrum of compound **9** in the aromatic region in CD_3OD.

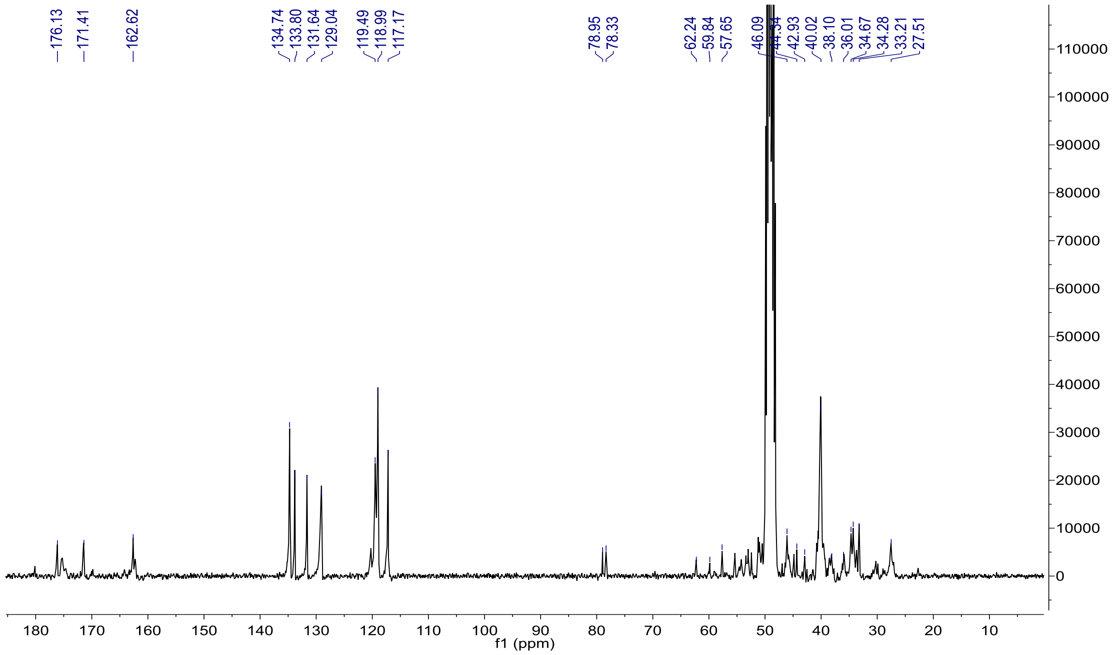

Figure 54. ^{13}C NMR spectrum of compound **9** in CD_3OD.

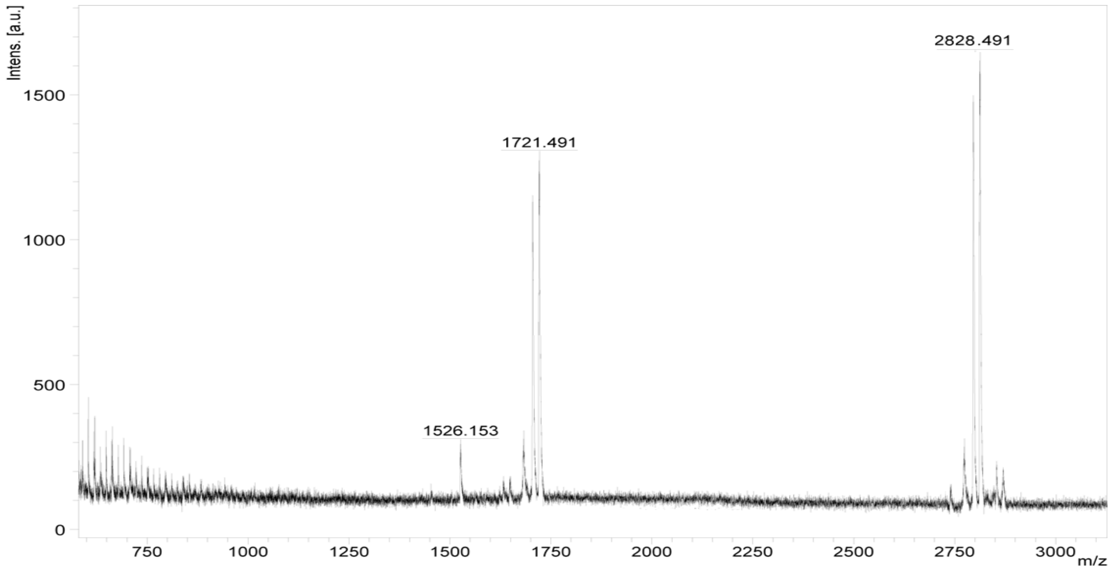

Figure 55. MALDI-TOF Mass spectrum of compound **9**.

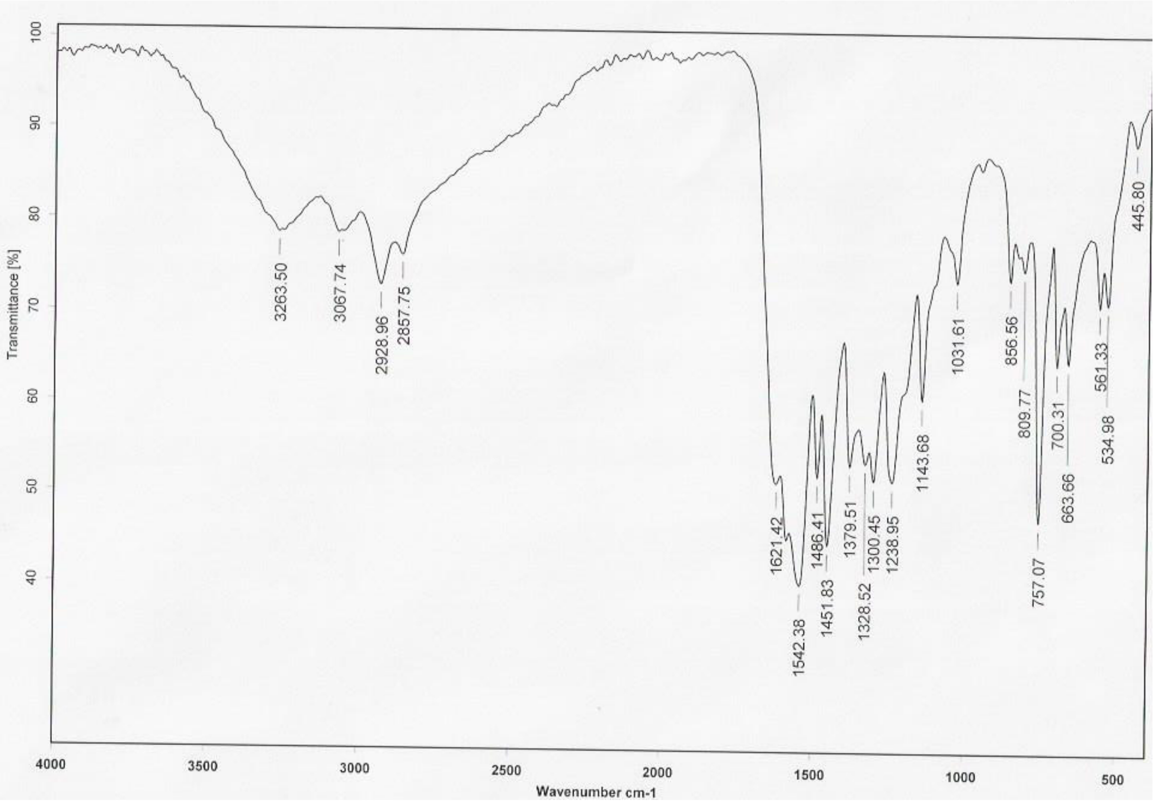

Figure 56. FTIR spectrum of compound **9**.

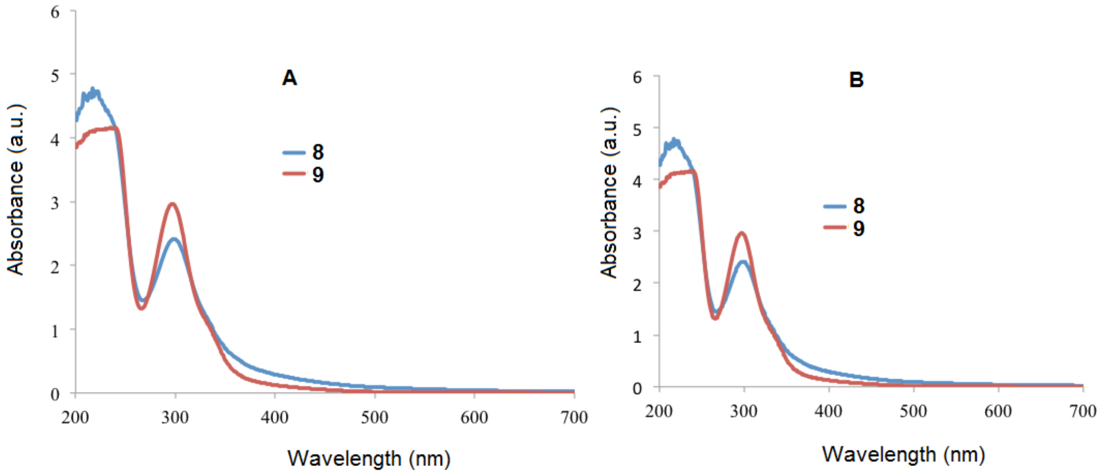

Figure 57. UV spectra of the compounds 8 and 9 at pH 1.2 M A) and at pH 7.4 B)
